# Technical Advance: Transcription factor, promoter, and enhancer utilization in human myeloid cells

**DOI:** 10.1189/jlb.6TA1014-477RR

**Published:** 2015-02-25

**Authors:** Anagha Joshi, Christopher Pooley, Tom C. Freeman, Andreas Lennartsson, Magda Babina, Christian Schmidl, Teunis Geijtenbeek, the FANTOM Consortium, Tom Michoel, Jessica Severin, Masayoshi Itoh, Timo Lassmann, Hideya Kawaji, Yoshihide Hayashizaki, Piero Carninci, Alistair R. R. Forrest, Michael Rehli, David A. Hume

**Affiliations:** *The Roslin Institute and Royal (Dick) School of Veterinary Studies, The University of Edinburgh, Scotland, United Kingdom; ^#^RIKEN Preventive Medicine and Diagnosis Innovation Program, Tsurumi-ku, Yokohama, Japan; ^‖^RIKEN Center for Life Science Technologies, Division of Genomic Technologies, Tsurumi-ku, Yokohama, Japan; ^†^Department of Biosciences and Nutrition, Karolinska Institute, Huddinge, Sweden; ^‡^Department of Dermatology and Allergy, Charité Universitätsmedizin Berlin, Germany; ^§^Department of Internal Medicine III, University Hospital, University of Regensburg, Germany; ^¶^Department of Experimental Immunology, Academic Medical Center, Amsterdam, the Netherlands; and **RIKEN Omics Science Center, Tsurumi-ku, Yokohama, Japan

**Keywords:** transcriptome, CAGE, hematopoiesis

## Abstract

The generation of myeloid cells from their progenitors is regulated at the level of transcription by combinatorial control of key transcription factors influencing cell-fate choice. To unravel the global dynamics of this process at the transcript level, we generated transcription profiles for 91 human cell types of myeloid origin by use of CAGE profiling. The CAGE sequencing of these samples has allowed us to investigate diverse aspects of transcription control during myelopoiesis, such as identification of novel transcription factors, miRNAs, and noncoding RNAs specific to the myeloid lineage. We further reconstructed a transcription regulatory network by clustering coexpressed transcripts and associating them with enriched cis-regulatory motifs. With the use of the bidirectional expression as a proxy for enhancers, we predicted over 2000 novel enhancers, including an enhancer 38 kb downstream of *IRF8* and an intronic enhancer in the *KIT* gene locus. Finally, we highlighted relevance of these data to dissect transcription dynamics during progressive maturation of granulocyte precursors. A multifaceted analysis of the myeloid transcriptome is made available (www.myeloidome.roslin.ed.ac.uk). This high-quality dataset provides a powerful resource to study transcriptional regulation during myelopoiesis and to infer the likely functions of unannotated genes in human innate immunity.

## Abstract (precis)

Analysis of the myeloid transcriptome by integrating 91 samples from the myeloid lineage and AML cell lines to predict novel regulatory interactions, enhancers, miRNAs, and lincRNAs.

## Introduction

Myeloid cells are a family of innate immune cells derived from pluripotent stem cells via a common, committed progenitor. They differentiate to become innate immune effector cells in response to various lineage-restricted growth factors, including CSF-1, GM-CSF, IL-3, IL-34, FLT3, and stem cell factor [1]. Each myeloid cell type has a distinct function and a set of gene products facilitating that function. Many genome-scale expression datasets have been generated in mouse and human systems to define the genes that distinguish different myeloid cell types [2, 3] and the underlying transcriptional regulatory networks [4, 5]. For example, the gene-expression profiles of human hematopoietic lineages, including progenitor populations, highlighted transcription modules that distinguish the broad erythroid, myeloid, and lymphoid classes [4]. We have generated related data based on meta analysis of human microarray data in the public domain [6].

Microarray-based data enable expression characterization at the gene level and not at the transcript level. To overcome this limitation, the FANTOM5 consortium used CAGE to assess promoter use in numerous primary cells and tissues, including progenitor and mature myeloid cell populations. CAGE tag sequencing produces a transcript-specific expression proxy enabling characterization of alternate promoter use [7]. Accordingly, transcription factors with multiple distinct promoters and distinct functional isoforms can be associated with their likely targets. Many widely expressed genes may still have specific functions in myeloid lineage divergence or cell-specific function. For example, differences in expression of metabolic enzymes distinguish subsets of human monocytes [8]. CAGE sequencing also detects products of the bidirectional promoter activity of active enhancers [9]. In this paper, a subset of 91 samples was selected from the FANTOM5 dataset (975 samples) [10], and we have dissected the myeloid cell populations separately to identify coregulated sets of promoters, novel enhancers, miRNAs, and noncoding RNAs specific to myeloid lineages. We highlight the use of these data to dissect transcript dynamics during progressive maturation of granulocyte precursors. The data reveal a much greater level of transcriptional complexity underlying myeloid lineage divergence than the classic binary view [11]. This work is part of the FANTOM5 project. Data download, genomic tools, details of the CAGE library generation and clustering, and copublished manuscripts are available at http://fantom.gsc.riken.jp/5/.

## MATERIALS AND METHODS

Ninety-one myeloid cell types were selected from the entire FANTOM5 dataset submitted to the DNA Data Bank of Japan database. They contain 18 distinct cell types in the myeloid lineage with at least 2 biologic replicates for each cell type and 22 AML samples. A description of sample preparation, quality control, and data processing is provided in Supplemental Methods. Supplemental Table 1 provides the details of each sample, as well as sequencing details, including number of TSSs identified in each sample. In brief, CAGE tags for each library were aligned to the genome (hg19), neighboring tags were grouped into clusters, and further individual TSSs were identified by use of decomposition-based peak identifications [10]. The TSSs were annotated based on known transcript 5′ ends within 500 bases and summarized into regions. The TSSs with at least 5 tags/million were selected for further analysis (details in Supplemental materials). Coexpression visualization and cluster analysis were performed by use of BioLayout *Express*^3D^ [12]. Initially, a sample-to-sample Pearson correlation matrix was calculated, graphs were visualized at a Pearson correlation cut-off value of 0.58 (see Fig. 2), and nodes were colored according to cell-type grouping to describe the similarity relations between samples. To identify loose relationships between samples, a heatmap of sample-to-sample Pearson’s correlation coefficients was also generated (Supplemental Fig. 1). Following this, a TSS level analysis was performed. A Pearson correlation matrix was calculated, comparing the expression profiles of the 106,709 TSSs, and a correlation cutoff, r = 0.9, was used to construct a graph. The resultant graph (nodes = 34,868) was then clustered by use of Markov cluster algorithm [13] with an inflation value of 2.2. Clusters with at least 20 tags and with 5 or more genes were selected for an in-depth analysis. To gain insights into the transcriptional mechanisms behind the control of these clusters, we performed cis-regulatory motif enrichment analysis by use of JASPAR [14, 15] motifs and HOMER [16]. We also calculated enrichment of each cluster with respect to the genome-wide binding patterns of transcription factors from ENCODE. Bidirectional expression was used as a proxy for predicting enhancer elements (details in Supplemental Methods). The functional enrichment was computed by use of a hypergeometric test with false discovery rate correction.

To facilitate analysis of the myeloid systems described in this manuscript, we have made the dataset available at www.myeloidome.roslin.ed.ac.uk. This web portal consists of 7 analysis tabs, namely, samples, clusters, genome browser, enhancers, miRNAs, lincRNAs, and granulopoiesis (explained in detail in the sections to follow). Each analysis tab highlights 1 aspect of use of these data. For example, the "clusters" tab lists the TSSs and corresponding genes in each cluster along with cis-regulatory control, whereas the "enhancers" tab provides cell type-specific enhancer locations and associated gene predictions. The "genome browser" tab provides a visualization of the myeloid sample data in the ZENBU genome browser [17], enabling users to view the expression of a peak across the 91 myeloid samples. The ZENBU genome browser system was developed for the FANTOM5 project to enable the interactive exploration of the FANTOM5 data sets. ZENBU can perform data-processing manipulations to allow the CAGE data to be visualized in many different ways.

## RESULTS

### Transcriptional profiling during normal and malignant myelopoiesis

To analyze the events in myeloid differentiation, we identified a total of 91 progenitor and mature myeloid primary cell populations and AML cell-line samples from the FANTOM5 dataset. Eighteen distinct cell types in myeloid lineage with at least 2 biologic replicates for each cell type and 22 AML samples were then analyzed by use of CAGE to assess promoter use during myelopoiesis. On average, 4 million CAGE tags mapped to hg19 assembly in each sample (Supplemental Table 1), and ∼7000 TSSs were identified for each sample.

The sample identity was consistent with expression of known lineage-specific growth regulatory receptors elaborated further with examples. *CSF1R* encodes the receptor for the macrophage growth and differentiation factor, M-CSF (CSF1). *CSF1R* gene transcription in myeloid cells initiated from a broad, purine-rich proximal promoter region with low but detectable expression in purified HPCs, and in CMPs. *CSF1R* transcription was induced in the GMPs, and increased in the transition from classic (CD14^+^, CD16^−^) to nonclassic (CD14^−^, CD16^+^) monocytes. *CSF1R* was highly expressed in immature LCs isolated from skin {validated by their strong expression of *CD207* (langerin) and *CD1a* [18]} and the monocyte-derived DCs. In contrast, mature LCs isolated from afferent lymph (CD207^−^ and CD1a^−^) and PDCs lacked detectable *CSF1R* and instead, expressed *FLT3*. The absence of *CSF1R* from the PDC in humans contrasts with its functional role in PDC in mice [19]. The FANTOM5 data confirm the existence of an alternative *CSF1R* TSS within the 3′ UTR of the upstream platelet-derived growth factor type *β*-type locus, used specifically in trophoblast and placental libraries [20] (data not shown). Across the dataset, *CSF1R* expression varies inversely with expression of *FLT3*, consistent with proposed roles of the 2 factors in lineage divergence between monocyte-macrophages and classic DCs [21]. In addition to the mature LCs and the PDCs, *FLT3* mRNA was expressed by HPCs and committed GMPs and in several AML lines, consistent with its involvement in myeloid leukemia [22] (Supplemental Fig. 1). The G-CSFR (*CSF3R*) was highly expressed by circulating neutrophils and eosinophils. Neutrophil PMN samples were purified from bone marrow and expressed much lower levels of *CSF3R* mRNA, implying a large increase in receptor expression as mature cells enter the circulation. Interestingly, *CSF3R* was also expressed by the classic CD14^+^, CD16^−^ monocytes but almost ablated in the CD16^+^ nonclassic subset (Supplemental Fig. 2). Finally, *CD117* (KIT), a surface marker of HPCs, was expressed in HPCs, CMPs, and GMPs. It was massively expressed in mast cells, and contrary to reports in the mouse [23], there was minimal expression in DCs or LCs (Supplemental Fig. 3). As expected, given the known role of IL-5 in allergy [24], the mast cells also specifically expressed high levels of the IL-5R. The *EMR1* gene product, recognized by mAb F4/80, is widely used as a macrophage differentiation marker in mice [25]. Recent reports have claimed that its expression is restricted to eosinophils in humans [26]. However, in the FANTOM5 dataset, *EMR1* transcription was detected in monocytes but not in eosinophils. It was elevated in the CD16^+^ monocyte subset, but by contrast to the mouse, where it is CSF1 inducible [25], it was ablated in all of the culture-derived macrophage/DC populations.

**Figure 1.**
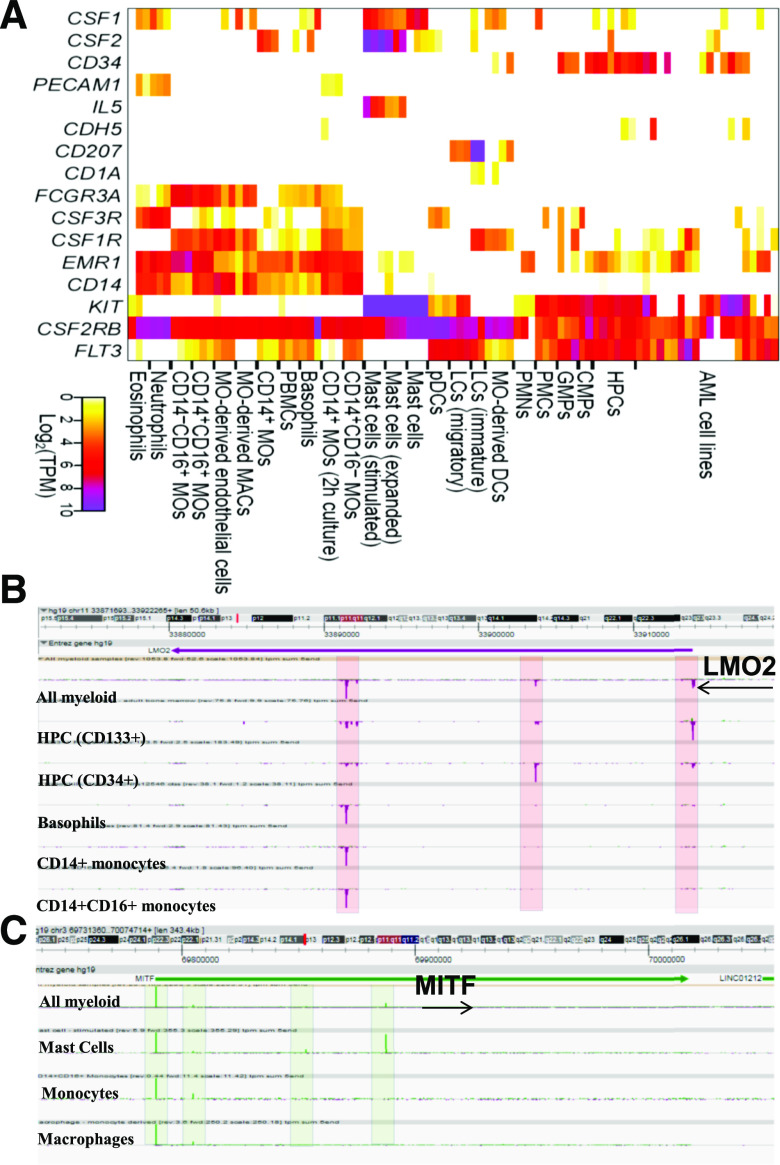
Expression of key surface markers and transcription factors across myeloid lineage. (A) Expression [log2 transcripts per million (TPM)] of key surface markers and transcription factors across 91 myeloid samples. (B) Normalized CAGE tags in the LMO2 gene locus in the ZENBU genome browser, highlighting differential expression of the 3 alternate transcripts in myeloid lineage. (C) Normalized CAGE tags in the MITF gene locus in the ZENBU genome browser with 4 alternate TSSs.

In summary, expression profiling of known key surface markers and regulators provides confidence on the identity of the cells (**Fig. 1A**) and the biologic relevance of downstream analysis.

### Alternate promoter use is a common feature of transcription regulators

CAGE-based expression profiling measures expression at multiple promoters in the same gene locus. We selected TSSs with more than 5 tags/million in at least 1 sample, resulting in a total of 106,709 TSSs (from a total of 3.5 million TSSs across 975 samples in the FANTOM5 dataset). Of these, 39,913 (40%) mapped to promoter regions identified by GENCODE 19, and 14,755 (15%) were associated with intragenic regions. The TSSs in the promoter or the gene body were ascribed to that gene name. The distribution of alternate TSSs to number of genes shows an approximate power-law relationship with an average of 5 TSSs per gene. The 1500 genes with 10 or more TSSs are enriched for "transcription regulation" (*P* value: 4.0e-19), regulation of apoptosis, and cell death (*P* value: 4.2e-20). They include transcription factors, such as *FOSB*, *FOXP1*, and *KLF6*, and cytokines, such as *IL-8* and *IL-1B*.

In individual samples, on average, 8000 TSSs map to ∼6000 unique genes, with an average of 1.2 TSSs per gene. This fraction is significantly lower than the 5 TSSs per gene observed across multiple cell types (*P* < 2.2e-16, Fisher test), indicating that alternate TSSs are used to control cell type-specific functions. Eosinophils and neutrophils have the highest number of expressed TSSs (∼11,000) with a ratio of 1.8 TSSs/gene.

Many of the known transcription regulators of myeloid lineages, including *LMO2*, *RARA*, *CEBPE*, *FOXP1*, *MITF*, mothers against decapentaplegic homolog 2, *KLF6*, and ETS translocation-variant gene 6,, have several alternate TSSs, some of which alter the N terminus of the protein. For example, LMO2 protein is a T cell oncogene, reportedly expressed in blood and endothelial progenitors in mice [27]. The human *LMO2* gene was reported to generate 3 alternate transcripts: LMO2-a, -b, and -c (Fig. 1B). LMO2-a and -b encode for the same 158 aa protein but differ in the 5′ UTR length [28]. The 2 distal TSSs (LMO-a/-b) were detected in HPCs and the AML samples but differed in relative expression (Supplemental Fig. 4). They were undetectable in committed progenitors, CMP or GMP. The same promoters were used in several CML lines, including K562, included in the primary FANTOM5 dataset. The proximal *LMO2* promoter (LMO-2c), on the other hand, highlights a function in monocyte differentiation and lineage divergence. LMO-2c transcript was barely detectable in progenitor populations (CMP, GMP) and highly expressed in monocytes but not at all in granulocytes or LCs.

RARA is implicated in myeloid differentiation through its involvement in the well-characterized t(15;17) translocation, which produces a promyelocytic leukemia-RARA fusion protein in AML [29]. At least 5 distinct transcripts with differing 5′ ends have been identified so far, and all are supported by the CAGE data. The major TSS is induced in all mature myeloid cells compared with the low levels in progenitors. Three more proximal TSSs, as well as antisense transcription associated with intronic enhancers, are restricted to mature myeloid cells and induced in these cells compared with progenitors (CMP and GMP). The transcription factor CEBPE has 2 reported promoters, forming multiple isoforms, including an inhibitory one, regulated by alternate TSS and splicing [30]. The data herein identified at least 5 TSSs. However, they were detected only in the HL60 cell line and were not detected in any primary cell population. Interestingly, a previous report on the association of CEBPE mutations in humans with specific granule deficiency in neutrophils seems to involve ectopic overexpression and does not imply a physiologic function [31].

Monocytes and macrophages in the mouse express all 4 members of the MITF family (*MITF*, *TFEC*, *TFEB*, and *TFE3*) [32], and these gene products have been implicated in regulating phagocyte-specific promoters associated with lysosomal hydrolases. All 4 members of the MITF family were differentially regulated in human myeloid cells from alternative promoters. MITF itself was expressed in differentiated macrophages and mast cells from the most distal of the 4 promoters (Fig. 1C). As reported in the mouse [21], TFEC is a monocyte-macrophage differentiation marker and absent from progenitors or granulocytes. These data confirm the existence of a 100 kb upstream TSS within the TFEC locus, also restricted to monocyte-macrophages. Finally, TFEB has several promoters, all of which were detected in myeloid cells. The expression was absent from stem cells and progenitors and induced in monocytes and granulocytes but not in mast cells. The MITF proteins can form heterodimers and homodimers with other MITF family members [33] and can interact with other lineage-specific factors, such as PU.1, so their complex expression pattern provides considerable scope for combinatorial function.

### Sample level analysis identifies signature gene sets in myeloid cells

**Figure 2.**
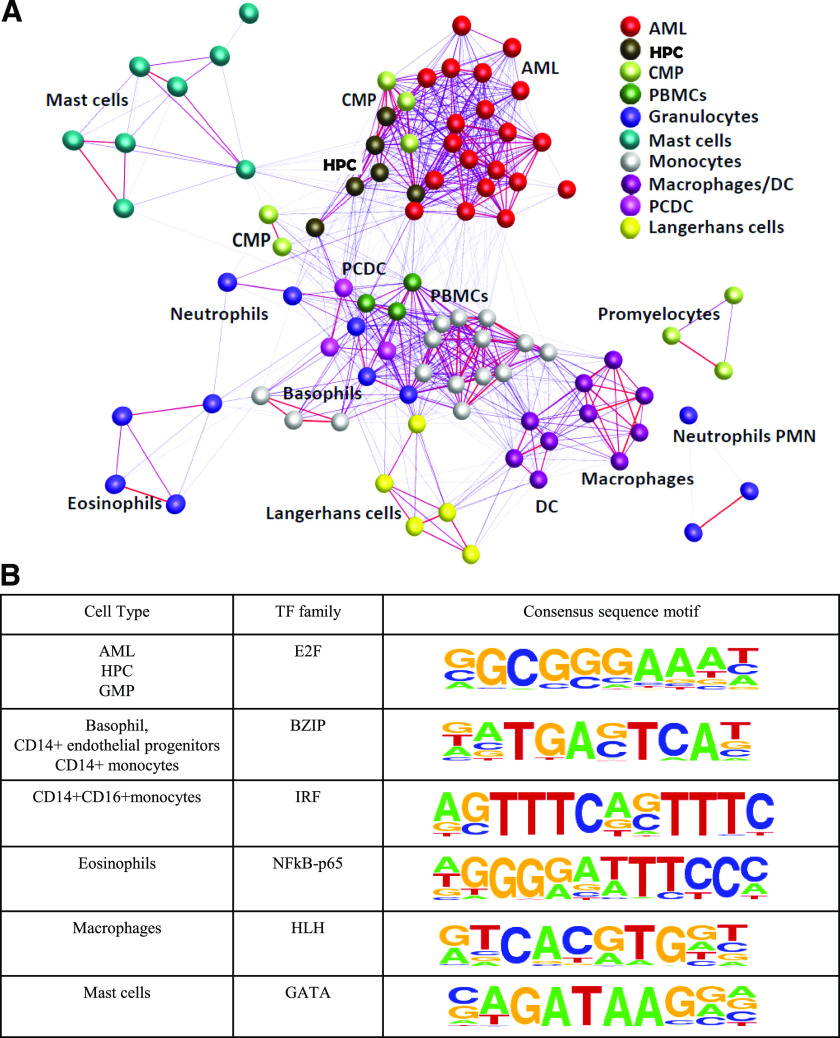
Sample clustering and cis-regulatory sequence motif enrichment. (A). Clustering and visualization of 91 myeloid samples with the Pearson correlation cutoff of 0.58 by use of BioLayout *Express*^3D^, highlighting the replicates as well as related sample clusters together. PCDC, plasmocytoid dentritic cells. (B) Cis-regulatory sequence motif enrichments along with sequence logos enriched only in specific cell type(s) calculated by use of HOMER software. TF, Transcription factor; BZIP, basic region-leucine zipper motif; HLH, helix-loop-helix.

To establish relatedness among the 91 samples, we created a sample-to-sample correlation matrix. The visual representation of this matrix at Pearson correlation coefficient cutoff of 0.58 by use of BioLayout *Express*^3D^ [12] shows that different samples of the same or related cell type, such as neutrophils and eosinophils, cluster together (**Fig. 2A**). Monocyte-derived macrophages (grown in CSF-1), DCs (grown in GM-CSF), and endothelial progenitors (grown in vascular endothelial growth factor) form 1 tight cluster. The monocyte samples cluster together regardless of the expression of CD16 or partial activation. The progenitor cells cluster together and lie closest to the AMLs, with which they share expression of cell cycle-related genes, such as proliferating cell nuclear antigen (data not shown). The dataset includes 3 mast cell populations, primary cells, and cells expanded in vitro and stimulated via the Fc*e*R1. Although they exhibit quite distinct gene-expression profiles [34], the cluster analysis reveals their relatedness to each other and distinguishes them from other myeloid populations.

Three sequence motifs (PU.1, GABPA, and TATA-binding protein) were enriched in all samples. PU.1 (SPI1) is an essential regulator controlling diverse aspects of early hematopoiesis and myeloid cell differentiation. The PU.1-expressing CMPs were found to have myeloid potential, whereas PU.1-deficient CMPs had erythroid potential, suggesting that PU.1 expression determines the developmental fate of a CMP [35]. Likewise, GABPA is also a key transcription factor required for myeloid differentiation [36]. Apart from these globally enriched transcription factor motifs, 6 motif families were restricted to 1 or a few cell types (Fig. 2B). For example, the E2F motif is enriched in progenitor populations and AML, whereas the GATA motif is enriched only in mast cells. GATA-1 and -2 have long been known for their importance in mast cell development (mastopoiesis) and mast cell function (such as cytokine responses), where a multitude of mast cell marker genes (FCER1A, MS4A2, KIT, and IL-1RL1/ST2) depends on GATA-1 and/or -2 for their transcription. The "samples" analysis tab in the web browser provides the genes and TSSs in each of 19 cell types together with genes specifically up- and down-regulated in that cell type, along with the predicted transcription regulators in each sample.

### Clustering of coexpressed TSSs

**Figure 3.**
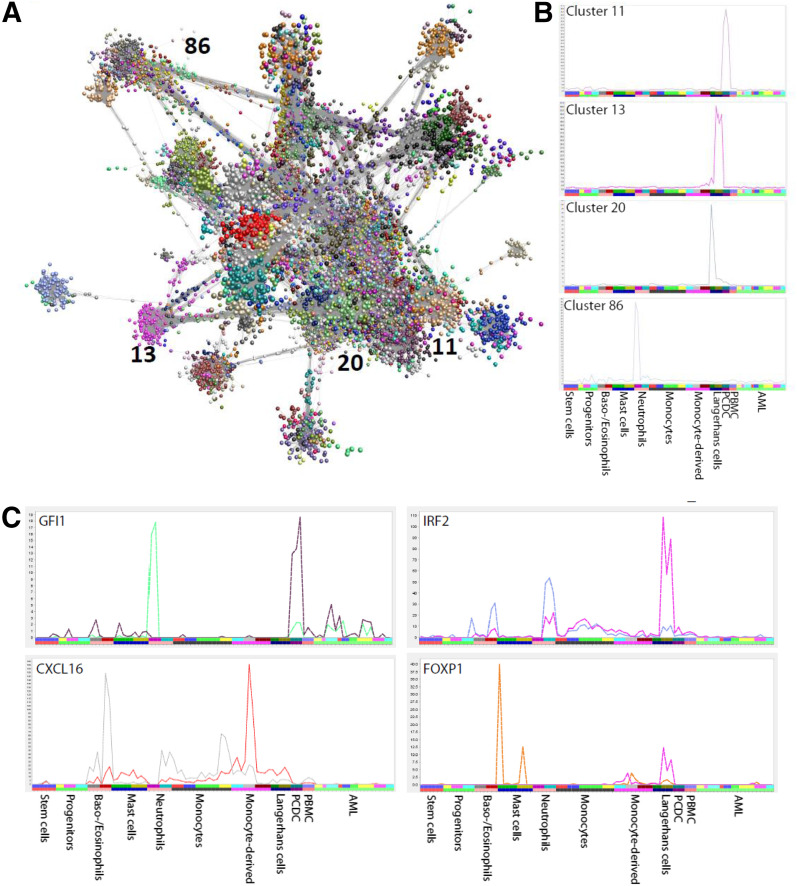
Gene clustering and alternate transcription regulation. (A) The clustering and visualization of all TSSs by use of BioLayout *Express*^3D^, with each of the TSSs belonging to each of 162 clusters indicated in a different color. (B) Expression profiles of 4 clusters (clusters 11, 13, 20, and 86) across 91 myeloid samples, indicating that many clusters are expressed in a specific cell type, providing a list of signature genes for that cell type, including distinction between immature and mature LCs (Clusters 13 and 20). (C) Four transcription factors with cell type-specific alternate transcripts across 91 cell types grouped into 11 subgroups labeled on the *x*-axis. The 2 (colored) profiles in each panel indicate 2 alternate transcripts expressed in distinct cell types. For example, Gfi1 places it into clusters 11 (expression profile in brown color) and 86 (expression profile in green color). Cluster 11 consists of genes highly expressed only in PDCs, whereas cluster 86 consists of genes highly expressed in neutrophils.

To identify sets of promoters that share transcriptional regulation [37], we used the clustering tool BioLayout *Express*^3D^ [12] to identify 162 clusters of coexpressed TSSs (**Fig. 3A**). Over 100 clusters contained TSSs highly expressed only in 1 or a few cell types (Supplemental Table 2). For example, cluster 1 was CD14^+^ monocyte specific, whereas cluster 2 was neutrophil and eosinophil specific. Cluster 68 distinguished LCs from other myeloid cell types, whereas clusters 13 and 20 further distinguished between the 2 LC populations (described in detail below). Several clusters contain genes highly expressed in a single AML sample, demonstrating that each AML sample has its unique signature genes. The clusters tab of the web browser provides the TSS positions, corresponding genes, predicted transcription regulators, as well as a graphical representation of the average expression dynamics for all clusters. We noted the presence of well-known transcription factors in multiple clusters, demonstrating the prevalence of use of alternate promoters to achieve cell type-specific gene regulation (Fig. 3B). For example, different promoter use by *GFI1* places it into 2 clusters: cluster 11 and 86 (Fig. 3C). Cluster 11 consists of genes highly expressed only in PDCs, whereas cluster 86 consists of genes highly expressed in neutrophils. With the use of the ENCODE chromatin immunoprecipitation sequencing data and cis-regulatory motif enrichment, we predict TCF family members to regulate cluster 11. TCF family members control developmental processes by forming homo- or heterodimers with other E proteins, such as the development of T cells, B cells, and PDCs at a transcriptional level [38]. TCF4 belongs to cluster 11.

As noted above, the LC populations are distinguished from each other and from monocyte-derived DCs by their relative expression of *CSF1R* and *FLT3*. The LCs contribute to 2 large clusters—13 and 20—that distinguish the LC from monocyte-derived DC and other cell populations (Fig. 3A). Both clusters contain *CD207* (langerin), expressed from distinct promoters. Cluster 13 contains a set of promoters highly enriched in the mature LC, lower in the “immature” LC, and largely absent from monocyte-derived DCs. The cluster includes *CCR7*, the central regulator of DC migration [39, 40]; the transcription factor *RELB*, also associated with DC function in humans [41]; as well as many class II MHC genes. Cluster 20 is enriched in the immature LC relative to migratory DC and interestingly, contains a distinct *CSF1R* promoter, as well as *CD1a*, many other class II MHC genes, and the transcription factor class II, MHC, transactivator.

### Enhancer analysis in the myeloid lineage predicts cell type-specific enhancers downstream of 2 key regulators: IRF8 and KIT

CAGE tag profiling detects candidate enhancers based on bidirectional transcription [9]. From the large FANTOM5 dataset, 20,301 enhancers were active in myeloid lineages (the complete list is available from the web resource). For the small number of genes studied in detail previously, the results confirm prior knowledge. For example, the data confirm the presence of a major myeloid-specific enhancer ∼16–17 kb upstream of the major TSS of PU.1 (SPI1), the location of a SNP that is more frequent in AML patients [42]. Supplemental Table 3 shows a further 10 of our enhancer regions that overlapped with a set of 2000 high-confidence enhancers identified by ENCODE [43]. Not surprisingly, as the enhancer predictions from ENCODE come from diverse sets of cell types, these 10 regions were active across multiple cell types and were associated with housekeeping genes and cell-cycle machinery, such as cell division protein kinase 6. Among the set of enhancers detected in myeloid cells, 196 were clearly restricted to a specific cell type. One-half of these was restricted to eosinophils and neutrophils. The genomic location, associated gene, as well as the expression profile across myeloid lineages of these putative enhancers are available from the enhancers tab of the web resource.

**Figure 4.**
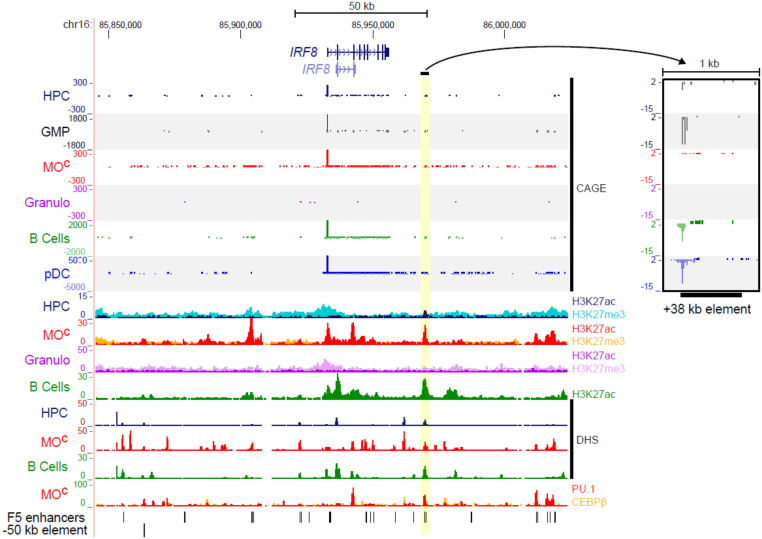
The +38 kb enhancer downstream of the IRF8 gene locus. Normalized CAGE tag counts in the IRF8 gene locus across selected myeloid samples with a zoom-in view of the putative +38 kb enhancer element associated with the IRF8 gene. The view also indicates peaks in this enhancer region for DHS, as well as H3K27ac (enhancer mark) in HPC and mature cell types. Moreover, there is PU.1 binding at the +38 kb enhancer region (the last track). MO^c^, monocytes.

One example of these lineage-specific enhancers is associated with *IRF8.* The *IRF8* mRNA was expressed in GMPs and maintained in monocytes. *IRF8* mutations in humans are associated with monocyte deficiency [44], including a SNP associated with variation in the monocyte count in humans [45]. It was induced further in PDCs but was switched completely off in granulocytes (Supplemental Fig. 5). With the reinforcement of the complex interactions of enhancers and promoters, at least 5 candidate enhancers are evident upstream, downstream, and within introns of the *IRF8* locus. We focused on a candidate enhancer region (chr16:85969764-85970393; hg19), specifically active in GMPs (**Fig. 4**). This enhancer is located 38 kb downstream of the *IRF8* gene TSS and was associated with *IRF8*, based on coexpression [9] (Fig. 4 and Supplemental Fig. 6A). In the FANTOM4 study of THP1 monocytic differentiation to macrophages in response to PMA, this enhancer was identified as a site of H3K9 acetylation based on whole genome tiling arrays [38]. The peak of H3K9 acetylation was reduced as IRF8 expression was extinguished in response to PMA. This region was the only one marked by H3K27Ac (a chromatin mark associated with active enhancers) in the otherwise repressed locus (as indicated by H3K27me3) in HPCs. It was strongly associated with H3K27Ac, DHS (indicating transcription factor binding), in *IRF8*-expressing monocytes and B cells and bound by PU.1 in monocytes (Fig. 4), a factor recently implicated in the remodeling of the *IRF8* locus [46] (Supplemental Fig. 6B). The candidate enhancer is also highly conserved between human and mouse (ecrbrowser.dcode.org) and also marked as a PU.1-bound enhancer in mouse macrophages (Supplemental Fig. 6C). The data outlined above indicate that this bidirectionally transcribed region acts as an enhancer that is most likely associated with transcriptional activation of the *IRF8* gene during hematopoietic differentiation. Another example is of an enhancer region chr4:55527478-55527674 (hg19), specific to mast cells, located 3.5 kb downstream of the main promoter of *KIT* (in the first intron) that shows an expression correlation with the *KIT* main promoter (Supplemental Fig. 7). This region is highly conserved across 100 vertebrates, including 2 highly conserved transcription factor-binding sites (Supplemental Fig. 7), supporting its role as a regulatory region. Moreover, this region is bound by *FOS*, *STAT3*, and *FOXP2* in the MCF10A-Er-Src cell line and contains a strongly enriched DNase-hypersensitive site from ENCODE data. This developmental gene locus has been characterized in great detail for cell type-specific chromatin looping [47].

### Transcription dynamics of miRNAs and noncoding RNAs in myeloid lineages

Many short, noncoding RNAs, in particular, miRNAs, act at the post-transcriptional level to control gene expression. Two hundred eleven miRNAs are expressed in myeloid lineages, including known regulators, during myelopoiesis. For example, miR-223 is preferentially expressed in myeloid cells and is involved in granulopoietic regulation [48]. lincRNAs also function in transcriptional regulation through several mechanisms. To identify robust, myeloid-specific lincRNAs, we first identified a set of CAGE-defined TSSs that do not lie within a gene body or promoter region of protein-coding genes, and 6286 of these were found to overlap with lincRNA locations from ENSEMBL. The list of miRNAs and lincRNAs expressed in myeloid lineages, along with their expression profile, is available from the "miRNAs" and "lincRNAs" tabs within the web resource, respectively.

To validate these datasets against current literature, we examined the HOX gene clusters, known for the prevalence of its intergenic, noncoding transcription. miRNAs and lincRNAs have been identified as regulators of HOX genes [49]. miRNA-196 was expressed specifically only in AML and progenitor cell populations (Supplemental Fig. 8), in keeping with evidence that miR-196a and miR-196b contribute to hematopoietic differentiation, proliferation, and AML [50]. On the 5′ and 3′ edges of the *HOXA* cluster, there are 2 lincRNA loci: the 3′ element called HOTAIRM1 [51] and the 5′ element called HOTTIP [52]. HOTAIRM1 is expressed specifically in myeloid lineage of the hematopoietic tree [51], and was detectably transcribed in all 91 samples (Supplemental Fig. 9). HOTTIP is a 3764 nucleotide, spliced, polyadenylated RNA, transcribed just upstream of *HOXA13*, and was expressed specifically in AML samples (Supplemental Fig. 10).

### Transcriptional dynamics during granulopoiesis

**Figure 5.**
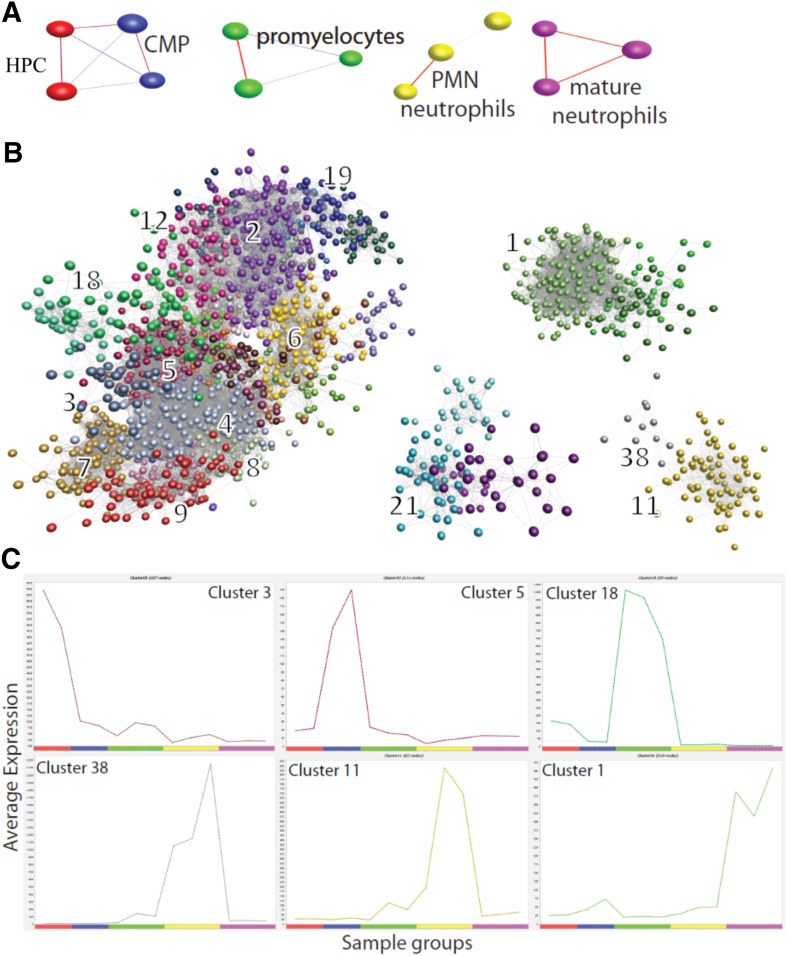
Temporal expression dynamics during granulopoiesis. (A) The clustering of samples and visualization of granulopoiesis by use of BioLayout *Express*^3D^ , highlighting the replicates that cluster together (HPC, red; CMP, blue; promyelocytes, green; PMN neutrophils, yellow; mature neutrophils, purple), and the samples follow the maturation/developmental order (from left to right) during granulopoiesis. (B) The clustering of genes and visualization of granulopoiesis by use of BioLayout *Express*^3D^, with a few exemplar clusters numbered. (C) The expression profile for 6 clusters: cluster 3, 5, 18, 38, 11, and 1, serially expressed during the granulopoiesis series.

The dataset provides a development of the granulocyte series, from HPCs through CMPs to promyelocytes and segmented neutrophils in the marrow and thence, to mature neutrophils in the circulation (**Fig. 5A**). We clustered 11,091 genes, dynamically expressed in the granulopoiesis series, to identify 38 sets of coexpressed gene sets. The expression clusters detected with CAGE are entirely consistent with the literature on progressive maturation of granulocytes [53] (Fig. 5B). We highlight this case by use of exemplar clusters that exhibit a clear, stage-specific expression profile (Fig. 5C). The first cluster (cluster 3) is HPC specific and proximal to cluster 4, which contains RUNX1 and T cell acute lymphocytic leukemia 1, the 2 transcription factors indispensable for generating HPCs [35]. The down-regulation of these stem cell-specific genes during granulopoiesis is consistent with the ability of RUNX1 to suppress myelopoiesis [35]. The second cluster (cluster 5), enriched in CD34^+^ progenitor cells, showed a functional enrichment for positive regulation of cell proliferation (*P* value: 1.4e-3) and contains transcription factors, such as JUN and NMYC. The third cluster (cluster 18) was enriched for mitochondrial matrix and lumen (*P* value: 7.7e-7) and contains the primary granule genes, such as myeloperoxidase, that are known to be highly expressed in GM progenitors, whereas the fourth cluster (cluster 38), enriched for response to microbicidal activity (*P* value: 1.7e-3), contains secondary granule proteins, such as lactoferrin, expressed in promyelocytes and marrow neutrophils and down-regulated in circulating neutrophils. The tertiary granule protein matrix metalloproteinase 9 and the components of the phagocyte oxidase, such as cytochrome B-245, are induced only at the marrow neutrophil stage and belong to the fifth cluster (cluster 11). Finally, chemotactic factor receptors, such as the formyl peptide receptor 1 and the complement receptor 1, are induced on blood neutrophils (as well as eosinophils, basophils, and CD14^+^ monocytes) and belong to the sixth cluster (cluster 1), enriched for inflammation response (*P* value: 1.4e-8) and myeloid leukocyte activation (*P* value: 1.2e-6). On the principle of guilt by association, other genes in these clusters, including those with little informative annotation, are likely to have functions in granulocyte maturation (for example, potential partners of the key transcription factors). Therefore, we have provided the lists of genes, including transcription factors and miRNAs, along with the graphical representation of the expression profile, for each cluster in the "granulopoiesis" tab of the web resource.

## DISCUSSION

Myeloid cells are essential players in innate immunity, derived from a common, committed progenitor. The FANTOM5 dataset includes hematopoietic progenitors and many distinct mature, primary human cell populations of myeloid origin, as well as diverse AML lines. We further validated sample identity by expression profiles of key surface markers, such as CSF1R, and CSF1R expression was largely excluded from various granulocyte lineages and mast cells. This finding contrasts with mouse granulocytes, where CSF1R mRNA (but not CSF1R protein) is highly expressed [54]. Nevertheless, many genes expressed outside of the myeloid lineages distinguish myeloid cell types and may use distinct promoters and enhancers. We have carried out a more-detailed analysis of this subset of FANTOM5 data, permitting the identification of novel transcripts, transcription factors, miRNAs, and noncoding RNAs specific to individual myeloid cell types that can be used as markers of differentiation. With the use of the bidirectional expression as a proxy for enhancers, we predicted over 2000 novel enhancers, including a +38 kb enhancer in the IRF8 and an intronic enhancer in the KIT gene locus. Finally, we highlighted relevance of these data to dissect transcription dynamics during progressive maturation of granulocyte precursors. We have made all analysis available to the research community through a web resource.

SNPs associated with human disease susceptibility in genome-wide association studies are associated with promoters and enhancers [9, 10]. Perhaps the greatest use of the FANTOM5 myeloid dataset is for a detailed (re)analysis of individual loci associated with human inflammatory diseases for hypothesis generation. For example, variation at the *NOD2* (caspase recruitment domain-containing protein 15) has been associated with susceptibility to Crohn’s disease [55]. The FANTOM5 data reveals that *NOD2* is monocyte specific, and expression is ablated in monocyte-derived macrophages. Interestingly, *NOD2* is located head to head with *SNX20*. A single report in the literature implicated SNX20 in P selectin-mediated myeloid cell adhesion [56]. There are 4 myeloid-specific enhancers between the 2 genes, and they are perfectly coexpressed. Hence, *SNX20* may contribute to Crohn’s disease susceptibility associated with variation at this locus.

Taken together, the high-quality dataset provides a powerful resource to study transcriptional regulation during myelopoiesis and to infer the likely functions of unannotated genes in human innate immunity.

## AUTHORSHIP

A.J. and D.A.H. designed the study, performed analysis, interpreted data, and wrote the manuscript. C.P. and J.S. developed the web resource. T.C.F. performed the analysis. T.C.F., M.R., and T.M. generated figures. The labs of M.B., A.L., T.G., M.R., and C.S. generated samples for CAGE profiling, M.I. produced the data. T.L. and H.K. performed CAGE data processing and management. A.R.R.F., P.C., and Y.H. were involved in the FANTOM5 concept, sample recruitment, and consortium management.

## Supplementary Material

Supplemental Data

## Abbreviations

AML: acute myeloid leukemia
CAGE: cap analysis of gene expression
CEBPE: CCAAT/enhancer-binding protein
CMP: common myeloid progenitor
DC: dendritic cell
DHS: DNase I hypersensitivity
EMR1: epidermal growth factor-like module-containing mucin-like hormone receptor-like 1
FANTOM: Functional Annotation of the Mammalian Genome
FLT3: fms-like tyrosine kinase-3
FOSB: FBJ murine osteosarcoma viraloncogene homolog B
FOXP1: forkhead box P3
GABPA: GA-binding protein *α*
GFI1: growth factor independence 1
GMP: granulocyte macrophage progenitor
H3K9/27: methylation of lysine 9/27 of histone 3
hg19: human genome version 19
HOTAIRM1: homeobox antisense intergenic RNA myeloid 1
HOTTIP: homeobox transcript at the distal tip
HOX: homeobox
HPC: hematopoietic progenitor cell
IRF: IFN regulatory factor
KIT: V-Kit Hardy-Zuckerman 4 feline sarcoma viral oncogene homolog
KLF6: Krueppel-like factor 6
LC: Langerhans cell
lincRNA: long, noncoding RNA
LMO2: LIM domain only 2
miRNA: microRNA
MITF: microphthalmia-associated transcription factor
NOD2: nucleotide-binding oligomerization domain 2
PDC: plasmacytoid dendritic cell
RARA: retinoic acid receptor *α*
RUNX1: runt-related transcription factor 1
SNP: single nucleotide polymorphism
SNX20: sorting nexin 20
SPI1: spleen focus-forming virus proviral integration oncogene 1
TCF: T lymphocyte chemotactic factor
TFEB/C/3: transcription factor EB/EC/E3
TSS: transcription start site
UTR: untranslated region
