# Supplementary material for: Technical Advance: Transcription factor, promoter, and enhancer utilization in human myeloid cells
Source: J Leukoc Biol. 2015 Feb 25;97(5):985–95. doi: 10.1189/jlb.6TA1014-477RR (PMC4398258; doi:10.1189/jlb.6TA1014-477RR)
Supplement: Supplemental Data [file supp_97_5_985__index.html]

Technical Advance: Transcription factor, promoter, and enhancer utilization in human myeloid cells — Supplemental Data 

# Technical Advance: Transcription factor, promoter, and enhancer utilization in human myeloid cells

## Supplemental Data

**Files in this Data Supplement:**

- Supplemental Data
- Supplemental Data
- Supplemental Data
- Supplemental Data
