# Supplementary material for: Technical Advance: Transcription factor, promoter, and enhancer utilization in human myeloid cells
Source: J Leukoc Biol. 2015 Feb 25;97(5):985–95. doi: 10.1189/jlb.6TA1014-477RR (PMC4398258; doi:10.1189/jlb.6TA1014-477RR)
Supplement: Supplemental Data [file supp_jlb.6TA1014-477RR_Supplemental_Data.docx]

Supplemental Material for

**Transcription factor, promoter and enhancer utilisation in human myeloid cells**

Anagha Joshi^1^¶, Christopher Pooley^1^, Tom Freeman^1^, Andreas Lennartsson^4^, Magda Babina^5^, Christian Schmidl^6^, Teunis Geijtenbeek^7^, the FANTOM consortium, Jessica Severin^3^, Masayoshi Itoh^3^, Timo Lassmann^3^, Hideya Kawaji^3^, Yoshihide Hayashizaki^2^, Harukazu Suzuki^3^, Alistair R. R. Forrest^3^, Michael Rehli^6^ and David Hume^1^¶

^1^The Roslin Institute and Royal (Dick) School of Veterinary Studies, The University of Edinburgh, Midlothian EH25 9RG, Scotland, United Kingdom

^2^RIKEN Preventive Medicine and Diagnosis Innovation Program, 1-7-22 Suehiro-cho, Tsurumi-ku, Yokohama, 230-0045 Japan.

^3^RIKEN Center for Life Science Technologies (Division of Genomic Technologies), 1-7-22 Suehiroo-cho, Tsurumi-ku, Yokohama, 230-0045 Japan.

^4^Department of Biosciences and Nutrition, Karolinska Institute, SE-14183 Huddinge, Sweden

^5^Department of Dermatology and Allergy, Charité Universitätsmedizin Berlin, Berlin, Germany

^6^Dept. Internal Medicine III, University Hospital, University of Regensburg, Germany

^7^Department of Experimental Immunology, Academic Medical Center, University of Amsterdam, Meibergdreef 9, 1105 AZ Amsterdam, the Netherlands.

¶To whom correspondence should be addressed

Running title: The Myeloid lineage transcriptome

**Supplementary methods:**

**Sample preparation for dendritic cells (DCs) and Langerhans cells (LCs):** Human tissues were obtained from healthy donors undergoing corrective breast or abdominal surgery after informed consent. The study was approved by Medical Ethics Review Committee in accordance with the ethical guidelines of the Academic Medical Center. Epidermal sheets were prepared as described previously^1^. Briefly, skin pieces were cut into 3-mm-thick pieces, containing the epidermis and dermis. The pieces were incubated with Dispase II (1 mg/ml, Roche Diagnostics) in Iscoves Modified Dulbecco’sMedium (IMDM), 10% FCS and gentamycine (10 mg/ml) for either 1 h at 37 C or overnight at 4 C. Epidermis was separated from dermis. LC-enriched epidermal single-cell suspensions were generated as described before [de Witte 2007]. Briefly, epidermal sheets were incubating in PBS containing DNase I (20 units/ml; Roche Applied Science) and trypsin (0.05% Beckton Dickinson) for 30 min at 37 C. Trypsin digestion was inactivated with FCS. Single-cell suspension was then layered on Ficoll gradient and immature LCs were purified using CD1a-labeled immunomagnetic microbeads (Miltenyi Biotec). Isolated LCs (99% CD1a+, langerin+) were tested for expression of HIV-1 related cell surface markers.

Mature LCs were obtained by culturing epidermis for three days. Migrated LCs were isolated and purified by CD1a positive selection using MACS, according to the manufacturer’s protocol (Miltenyi Biotec, Auburn, CA). This procedure yielded a >95% pure CD1a+ LC population. Monocytes were isolated by density centrifugation of PBMCs and cultured for five days in the presence of 800 U/ml IL-4 and 1000 U/ml GM-CSF to stimulate differentiation into DCs. The purity of obtained DCs was >90%.

**Sample preparation for Mast cells (MCs from Motakis et. al.**^2^**)**: The skin was obtained from cosmetic breast-reduction surgeries with informed consent of the patients. All experiments were conducted according to the Declaration of Helsinki Principles and approved by the ethics committee of the Charité Universitätsmedizin Berlin. Briefly, skin was cut into strips and treated with dispase (Becton Dickinson, Heidelberg, Germany) at 4°C overnight. After removal of the epidermis, the dermis was chopped into small pieces and digested with collagenase (Worthington, Lakewood, NJ), hyaluronidase (Sigma, Deisenhofen, Germany), and DNase I (Roche, Basel, Switzerland) for 1 hour at 37°C. After 3 steps of filtration, the remaining tissue was subjected to a second digestion step. MC purification from the dispersates was achieved by selection with anti–human c-Kit microbeads (Miltenyi Biotec, Bergisch Gladbach, Germany) and an automated magnetic-activated cell sorting separation device. Viability (trypan-blue exclusion) and purity (acidic toluidine-blue staining) exceeded 99%. The high degree of MC purity was confirmed in Motakis et. al.2, as MCs expressed all known MC markers at highest levels across the atlas (e.g. KIT, HDC, CMA1), while lineage markers of other skin cell subsets were entirely absent from the MC samples (THY1 for fibroblasts, KDR/CDH5 for endothelial cells, KRT10/IVL for keratinocytes, TYR for melanocytes, and CD1A/CD207 for Langerhans cells). A total of 0.8 to 1.1 3 10^7^ cells (from 1 donor) were immediately lysed in TRIzol and further processed for HeliScopeCAGE. MCs from 3 donors were used for the ex vivo analysis (donors 2, 3, and 4). To obtain the expanded samples, MCs were cultured for 4 to 5 weeks (donors 1, 5, and 8). Stimulation was achieved by FceRI crosslinking with AER-37 at 2 mg/mL (eBioscience, San Diego, CA). Only samples showing .60% of activation (degranulation; CD107a upregulation) were included as stimulated samples in the deep-CAGE analysis

**Other samples preparation:** The human primary cell samples were purchased as purified RNA from Cell Applications, 3HBiomedical or Sciencell. Additional primary cells were also purchased from Cell systems, CET, Lonza, Promocell, Sciencell, Stem cell technologies and Xenotech. These were cultured as per the manufacturer’s instructions, and then RNA extracted using the miRNeasy kit (QIAGEN). The cell lines used are all available from public repositories (RIKEN BRC (<http://www.brc.riken.jp/lab/cell/english/> ), ATCC (<http://www.atcc.org/> ), Coriell (<http://ccr.coriell.org/> ), ECACC (<http://www.hpacultures.org.uk/collections/ecacc.jsp> ), and Japan Health Sciences foundation - Health Science Research Resources Bank (<http://www.jhsf.or.jp/English/index_p.html)>). Briefly, frozen cell line stocks were rapidly thawed at 37^o^C, diluted in 10ml 37^o^C PBS, pelleted, and RNA directly extracted using the miRNA easy Kit. The purification and sorting of CMP, GMP and promyelocytes with FACS data and surface markers are described in Ronnerblad *et. al.*^3^ The purification and sorting of macrophages are described in Schroder *et.al.*^4^ The purification and sorting of monocytes are described in Schmidl *et. al.*^5^

**Quality control:** Working with large numbers of samples from multiple collaborators and companies brings about 3 potential issues of QC (RNA quality, library depth and sample identity). RNA Quality: Degraded RNA can affect the quality of CAGE libraries affecting both the promoter hit rate and the complexity of transcript species measured. To address this, RNA integrity measurements were made using an Agilent Bioanalyser for samples with more than 1ug of RNA available. Most of the samples used in the study had RIN above 6.8. For low quantity libraries this step was skipped so not to waste RNA and library quality metrics used instead. Library depth: shallow libraries can lead to false negative calls on gene expression. For the purposes of the gene expression analyses used in this paper libraries needed to contain at least 500,000 successfully aligned reads (mapping quality is 20 or more, and sequence identity is 85% or more) mapped tags. In addition the fraction of mapped tags falling within the robust peak regions were used as an additional metric for library quality. Sample identity: finally sample hierarchical clustering and marker gene checks were used to confirm the identity of samples.

**Data processing**: Sequenced Heliscope reads have a high sequencing error rate (~5%), vary in length and lack an estimation of base qualities. Combined these factors make the data processing challenging. As an initial step we removed reads corresponding to ribosomal RNA. We accomplish this by directly aligning each read against the whole human ribosomal DNA complete repeating unit and discarding all reads with an edit distance smaller or equal to two. For this purpose we implemented Myers’ bit parallel dynamic programming algorithm in the program rRNAdust (author: T. Lassmann)^6^. For computational efficiency we further parallelized this algorithm. All CAGE reads were mapped to the genome hg19 using Delve, a probabilistic mapper. After all error probabilities are estimated, individual reads are placed to a single position on the genome where the alignment has the highest probability to be true according to the pHMM model. Phred scaled mapping qualities, reflecting the likelihood of the alignment at a given genome position, are also reported. Reads mapping with a quality of less than 20 (<99% chance of true) were discarded. Furthermore, we discarded all reads that map to the genome with a sequence identity of less than 85%. We used samtools 1.4 to extract the raw data counts for each Transcription Start Site (TSS) from all FANTOM5 .bam files. Approximately, 4-16 million CAGE tags for each library were aligned to the genome (Hg19). For each TSS, we estimated a mapping quality score and only TSSs with < 1% mapping error were kept for further analysis. The quality mapped TSSs were grouped into CTSS (clusters of TSS with common start site). The CTSS data were the summarized counts from all TSS included in the cluster (summation per sample). A substantial fraction of the peaks identified above had very limited tag support and were located in exonic regions, while the majority of known transcript 5’ ends were well supported by peaks with many tags. To enrich for promoter associated signals, we examined thresholds in expression levels at individual single CTSSs, with the thesis that genuine TSS are likely to reproducibly use the same position, whereas random degradation should be spread more broadly along the transcript. We set this by examining the ratio of peaks that were near 5’ ends of known transcripts (within 500bp) versus peaks that were within internal exons (but not promoter). We settled on two thresholds the first a permissive threshold gave a ratio of promoter to exonic peaks of ~0.7 and corresponded to the subset of peaks with a single CTSS in a single experiment supported by 3 or more observations in at least one profile, and a robust threshold yielding a ratio of ~2.0 and corresponding to peaks with a single CTSS in a single experiment supported by 11 or more observations and 1 or more TPM. Although the thresholds are based on single nucleotide positions, the total number of observations (reads) in each CAGE peak is substantially more. Using the robust peaks defined above we counted tags which 5’ end alignments (mapping quality >=20, percent identity >= 85%) started within the boundaries of individual robust peaks. We counted the CAGE reads arising from individual CAGE peaks in each of the selected profiles and normalized the counts as TPM (tags per million) based on the library size and normalization factors estimated by edgeR using the relative log expression (RLE) method. To systematically annotate the DPIs based on their relationships with known genes and transcripts, we compared them to the following gene models downloaded from the UCSC Genome database January 2012: RefSeq, UCSC known gene, Gencode V7 transcripts, and full-length mRNA tracks. CAGE peaks were assigned to a gene or transcript if their 5’ end was on the same strand and within 500bp of the 5’ end of the transcript model. In this process, gene models whose 5’-ends do not correspond to transcription starting sites (e.g. snoRNA, snRNA, and miRNA 5’ ends result from cleavage of primary transcripts) were given lower priority. From the transcript and gene associations we further extended the annotation and provided HGNC gene symbols, EntrezGene IDs, and UniProt IDs (if coding) according to their association with the selected gene models. We extracted the CAGE tags for 91 myeloid lineage samples from the “robust” DPI peaks. The data consisted of 18 progenitor and mature cell types in myeloid lineage with 2 or 3 biological replicates for each cell type together with 22 acute myeloid leukaemia samples.

**Enhancer Prediction**: Bidirectionally transcribed loci were defined from forward and reverse strand CAGE tag clusters (TCs) supported by at least two CAGE tags in at least one sample. Only TCs not overlapping antisense TCs were used. A centre position was defined for each bidirectional locus as the mid position between the rightmost reverse strand TC and leftmost forward strand TC included in the merged bidirectional pair. Each bidirectional locus was further associated with two 200 bp regions immediately flanking the centre position, one (left) for reverse strand transcription and one (right) for forward strand transcription, in a divergent manner. The merged bidirectional pairs were further required to be bidirectionally transcribed (CAGE tags supporting both windows flanking the centre) in at least one individual sample, and to have a greater aggregate of reverse CAGE tags (over all FANTOM5 samples) than forward CAGE tags in the 200 bp region associated with reverse strand transcription, and vice versa. A directionality score, D, was defined for each enhancer over aggregated normalized reverse, R, and forward, F, strand expression values across all samples (Supplementary Fig. 6a); D = (F − R)/(F + R). D ranges between −1 and 1 and specifies the bias in expression to reverse and forward strand, respectively (D = 0 means 50% reverse and 50% forward strand expression, while |D| close to 1 indicates unidirectional transcription). Bidirectional loci were further filtered to have low, non-promoter-like, directionality scores (|D| < 0.8) and to be located distant to TSSs and exons of protein- and noncoding genes. Finally myeloid specific enhancers were selected which resulted in a final set of 20,301 putative enhancers.

**Supplementary Figure 1:** Normalized CAGE tag counts in the FLT3 gene locus for 91 myeloid samples in ZENBU genome browser^7^ (bottom) along with the sum of all tag counts from 91 samples displayed along the FLT3 gene (top).

**Supplementary Figure 2:** Normalized CAGE tag counts in the CSF3R gene locus for 91 myeloid samples in ZENBU genome browser^7^ (bottom) along with the sum of all tag counts from 91 samples displayed along the CSF3R gene (top).

**Supplementary Figure 3:** Normalized CAGE tag counts in the KIT gene locus for 91 myeloid samples in ZENBU genome browser^7^ (bottom) along with the sum of all tag counts from 91 samples displayed along the KIT gene (top).

**Supplementary Figure 4:** Normalized CAGE tags in the LMO2 gene locus for 91 myeloid samples in ZENBU genome browser^7^ highlight three alternate TSSs matching with 3 known LMO2 transcripts – LMO2a,b and c.

**Supplementary Figure 5:** Normalized CAGE tag counts in the IRF8 gene locus for 91 myeloid samples in ZENBU genome browser^7^ (bottom) along with the sum of all tag counts from 91 samples displayed along the IRF8 gene (top).

**Supplementary Figure 6:** Normalized CAGE tag counts at the +38kb enhancer elements downstream of IRF8 gene locus for 91 myeloid samples in ZENBU genome browser^7^ (top). The bidirectional tag counts in three GMP samples. H3K27ac peak from ENCODE data validating the enhancer element. The mouse homolog of +38kb enhancer is bound by Pu.1 (bottom).

**Supplementary Figure 7:** Normalized CAGE tag counts in the predicted enhancer region in the first intron of KIT gene for 91 myeloid samples in ZENBU genome browser^7^ (top). The same enhancer region in UCSC genome browser is conserved across 100 vertebrates and is a region marked in DNase assays from the ENCODE project.

**Supplementary Figure 8:** Normalized CAGE tag counts in the mir-196 miRNA locus for 91 myeloid samples in ZENBU genome browser^7^ (bottom) along with the sum of all tag counts from 91 samples and for acute myeloid leukaemia samples displayed along the mir-196 miRNA gene (top).

**Supplementary Figure 9:** Normalized CAGE tag counts in the HOTAIRM1 lincRNA locus for 91 myeloid samples in ZENBU genome browser^7^ (bottom) along with the sum of all tag counts from 91 samples displayed along the HOTAIRM1 lincRNA (top).

**Supplementary Figure 10:** Normalized CAGE tag counts in the HOTTIP lincRNA locus for 91 myeloid samples in ZENBU genome browser^7^ (bottom) along with the sum of all tag counts in acute myeloid leukaemia samples displayed along the HOTTIP lincRNA (top).

**Supplementary Figure 11:** A heatmap of sample-to-sample Pearson’s correlation coefficients highlighting the replicates as well as related samples cluster together.

References:

1. De Witte L, Nabatov A, Pion M, et al. Langerin is a natural barrier to HIV-1 transmission by Langerhans cells. *Nat. Med.*. 2007;13(3):367–371.

2. Motakis E, Guhl S, Ishizu Y, et al. Redefinition of the human mast cell transcriptome by deep-CAGE sequencing. *Blood*. 2014;123(17):e58–67.

3. Rönnerblad M, Andersson R, Olofsson T, et al. Analysis of the DNA methylome and transcriptome in granulopoiesis reveals timed changes and dynamic enhancer methylation. *Blood*. 2014;123(17):e79–89.

4. Schroder K, Irvine KM, Taylor MS, et al. Conservation and divergence in Toll-like receptor 4-regulated gene expression in primary human versus mouse macrophages. *Proc. Natl. Acad. Sci. U. S. A.*. 2012;109(16):E944–953.

5. Schmidl C, Renner K, Peter K, et al. Transcription and enhancer profiling in human monocyte subsets. *Blood*. 2014;123(17):e90–e99.

6. FANTOM Consortium and the RIKEN PMI and CLST (DGT), Forrest ARR, Kawaji H, et al. A promoter-level mammalian expression atlas. *Nature*. 2014;507(7493):462–470.

7. Severin J, Lizio M, Harshbarger J, et al. Interactive visualization and analysis of large-scale sequencing datasets using ZENBU. *Nat. Biotechnol.*. 2014;32(3):217–219.

8. Kheradpour P, Ernst J, Melnikov A, et al. Systematic dissection of regulatory motifs in 2000 predicted human enhancers using a massively parallel reporter assay. *Genome Res.*. 2013;23(5):800–811.
