# Supplementary material for: Technical Advance: Transcription factor, promoter, and enhancer utilization in human myeloid cells
Source: J Leukoc Biol. 2015 Feb 25;97(5):985–95. doi: 10.1189/jlb.6TA1014-477RR (PMC4398258; doi:10.1189/jlb.6TA1014-477RR)
Supplement: Supplemental Data [file supp_jlb.6TA1014-477RR_Supplemental_Figures.pptx]

## Slide 1
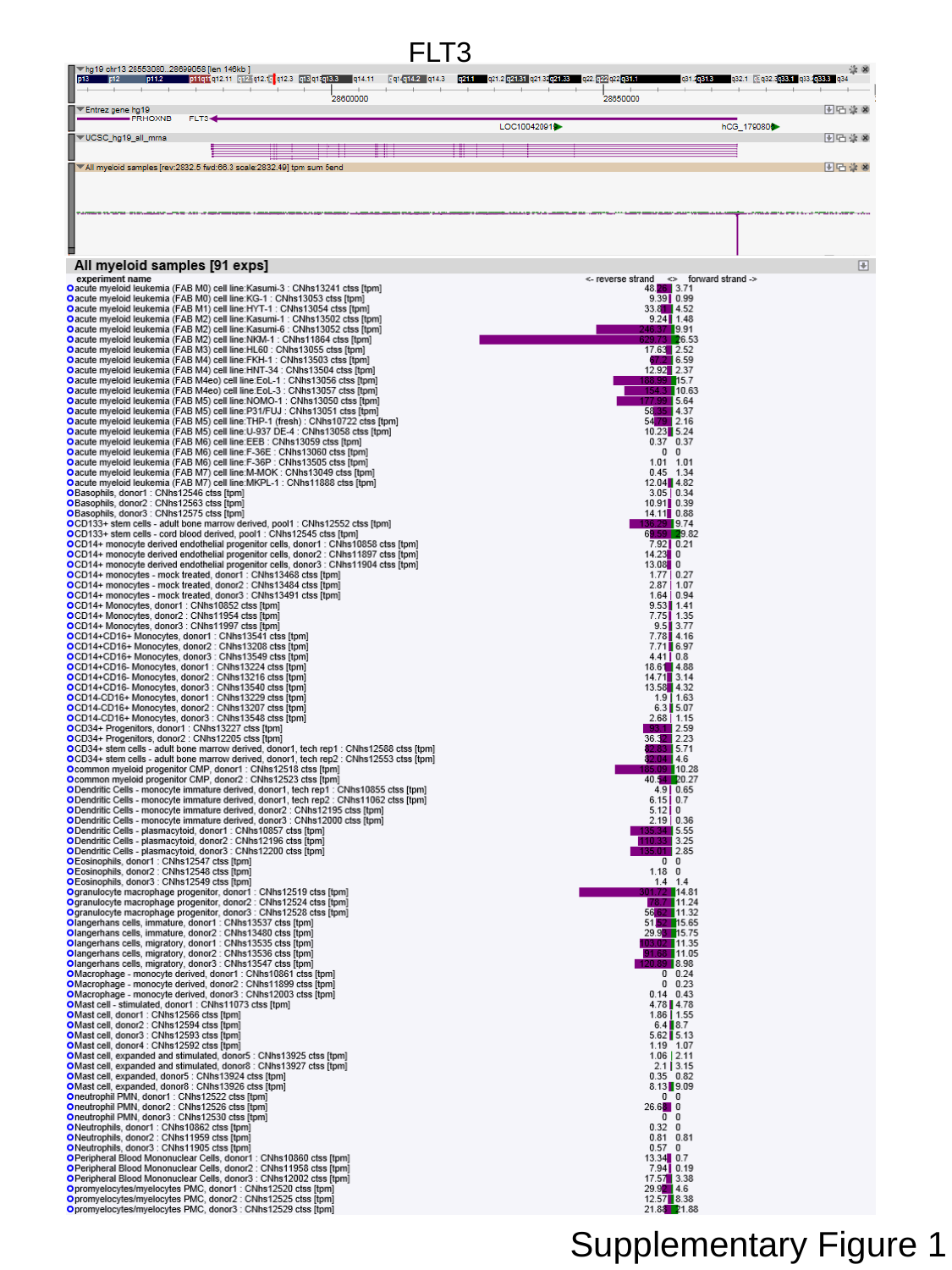

FLT3
Supplementary Figure 1

## Slide 2
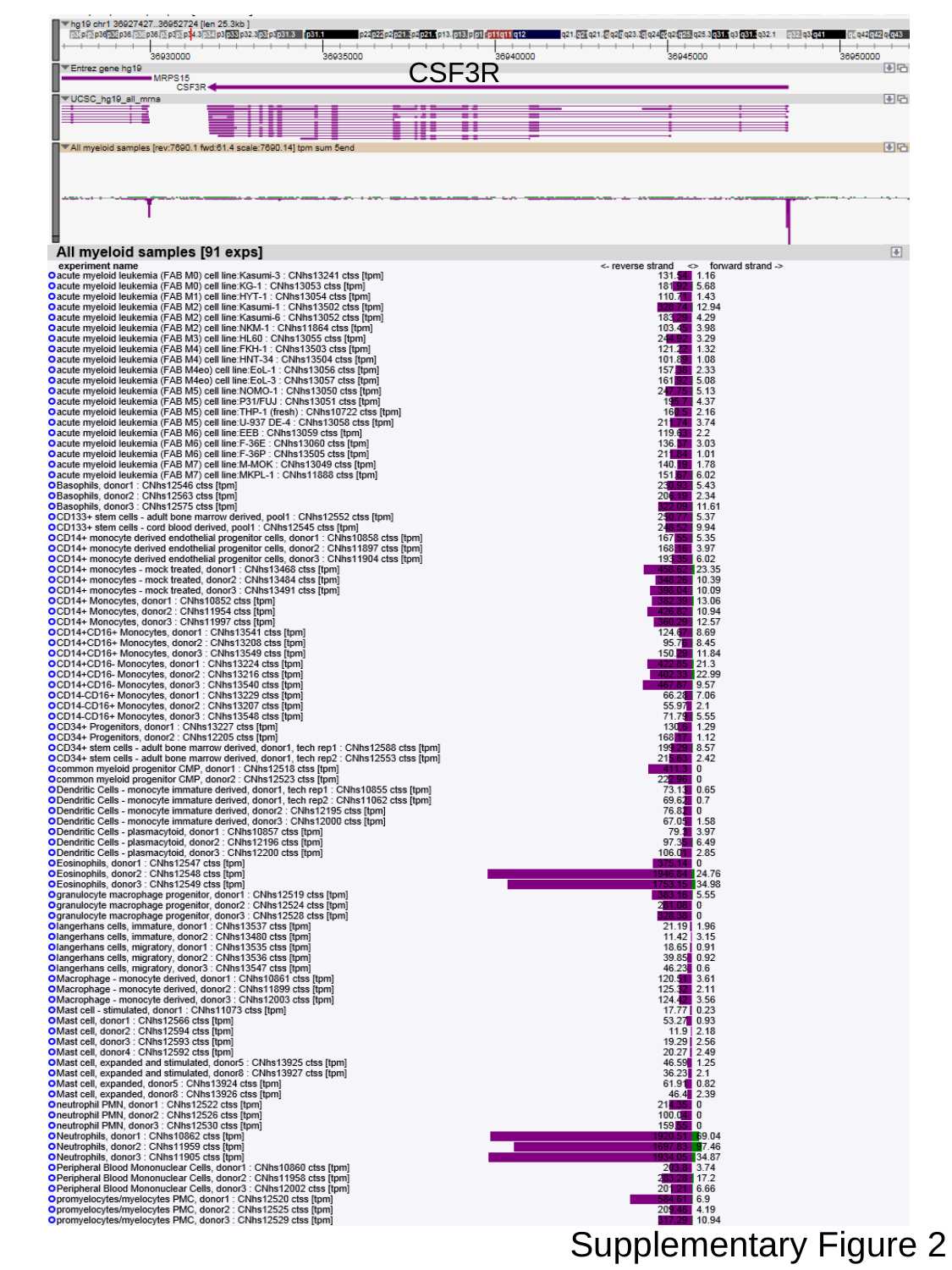

CSF3R
Supplementary Figure 2

## Slide 3
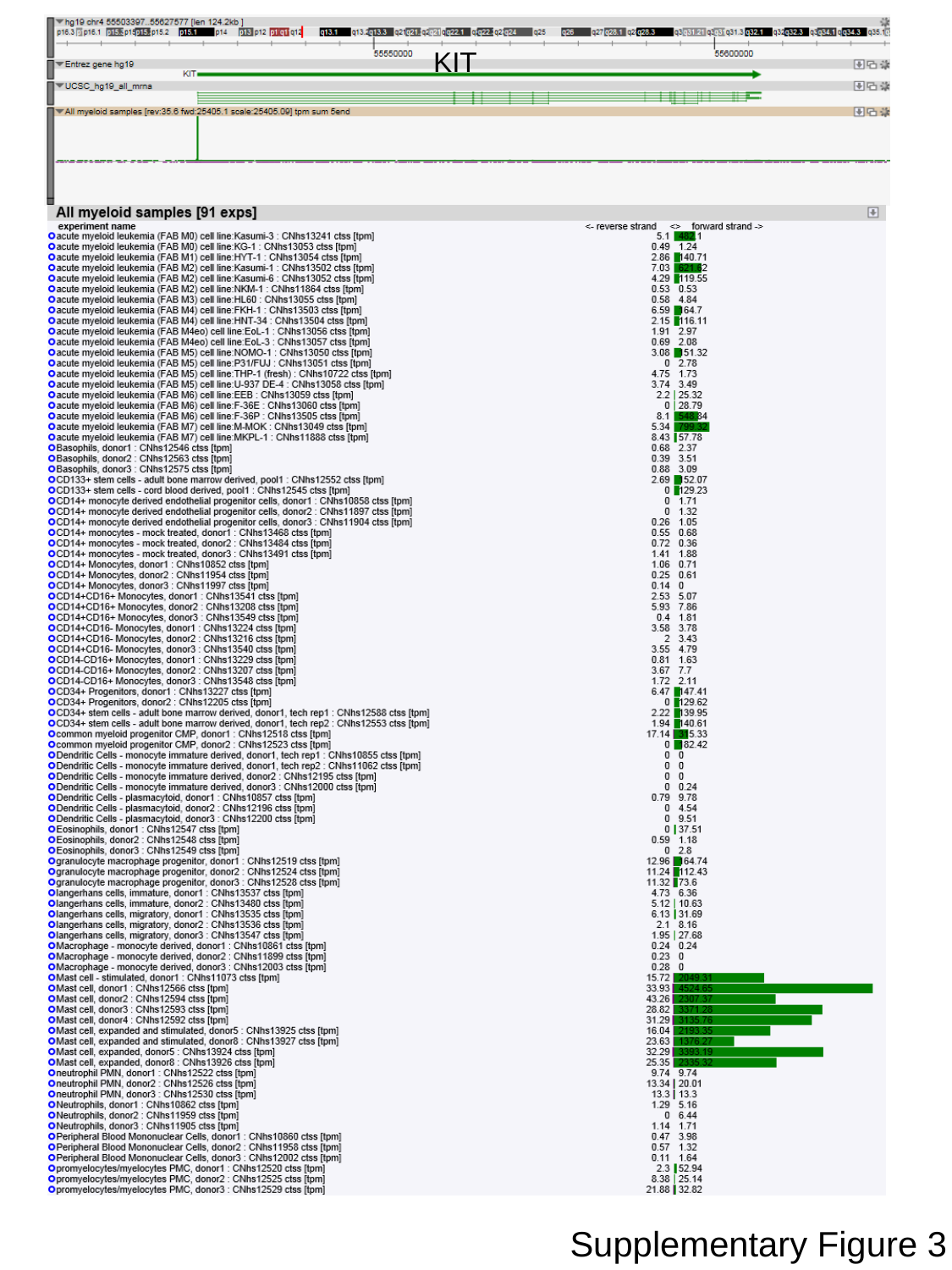

KIT
Supplementary Figure 3

## Slide 4
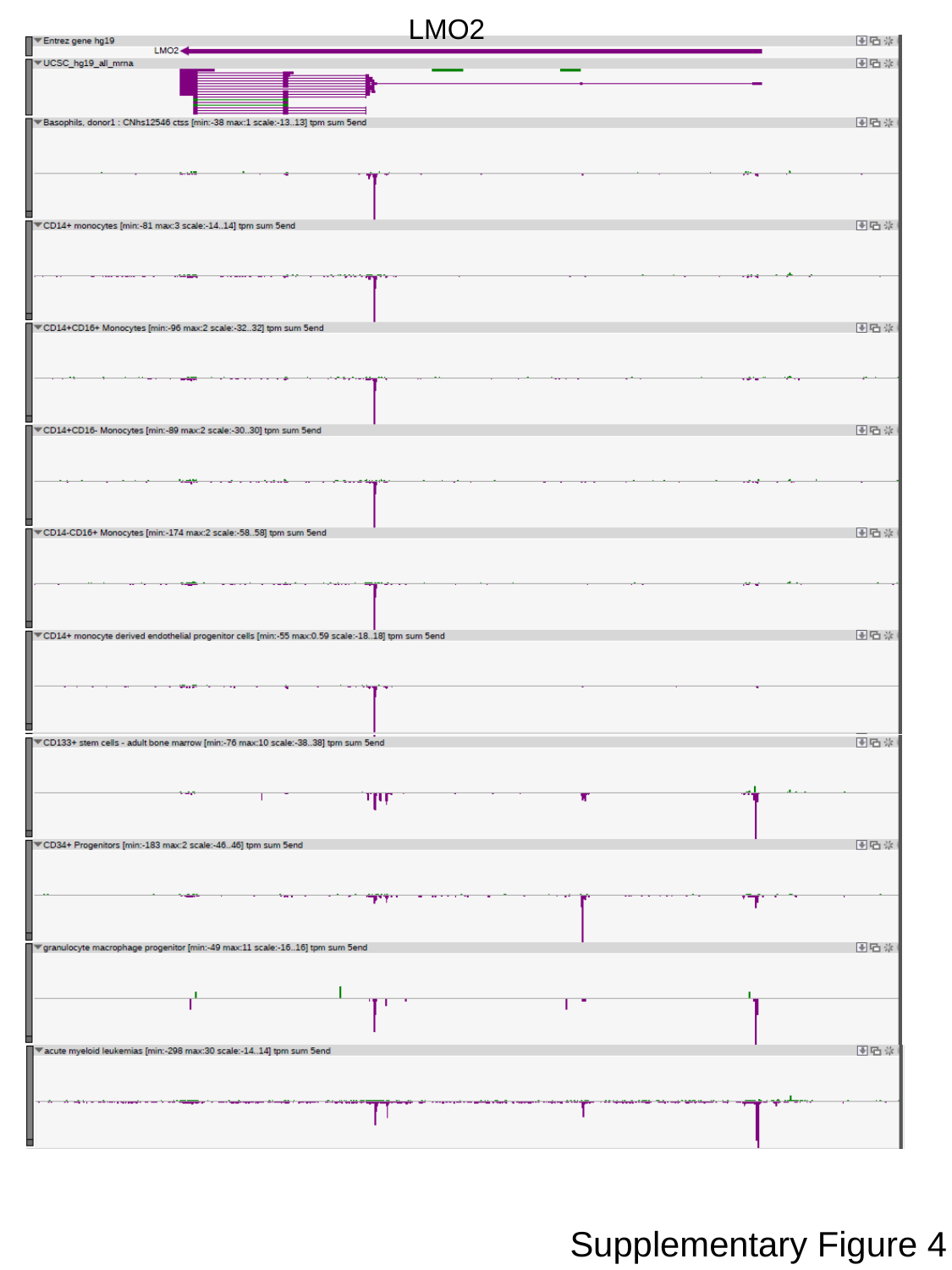

LMO2
Supplementary Figure 4

## Slide 5
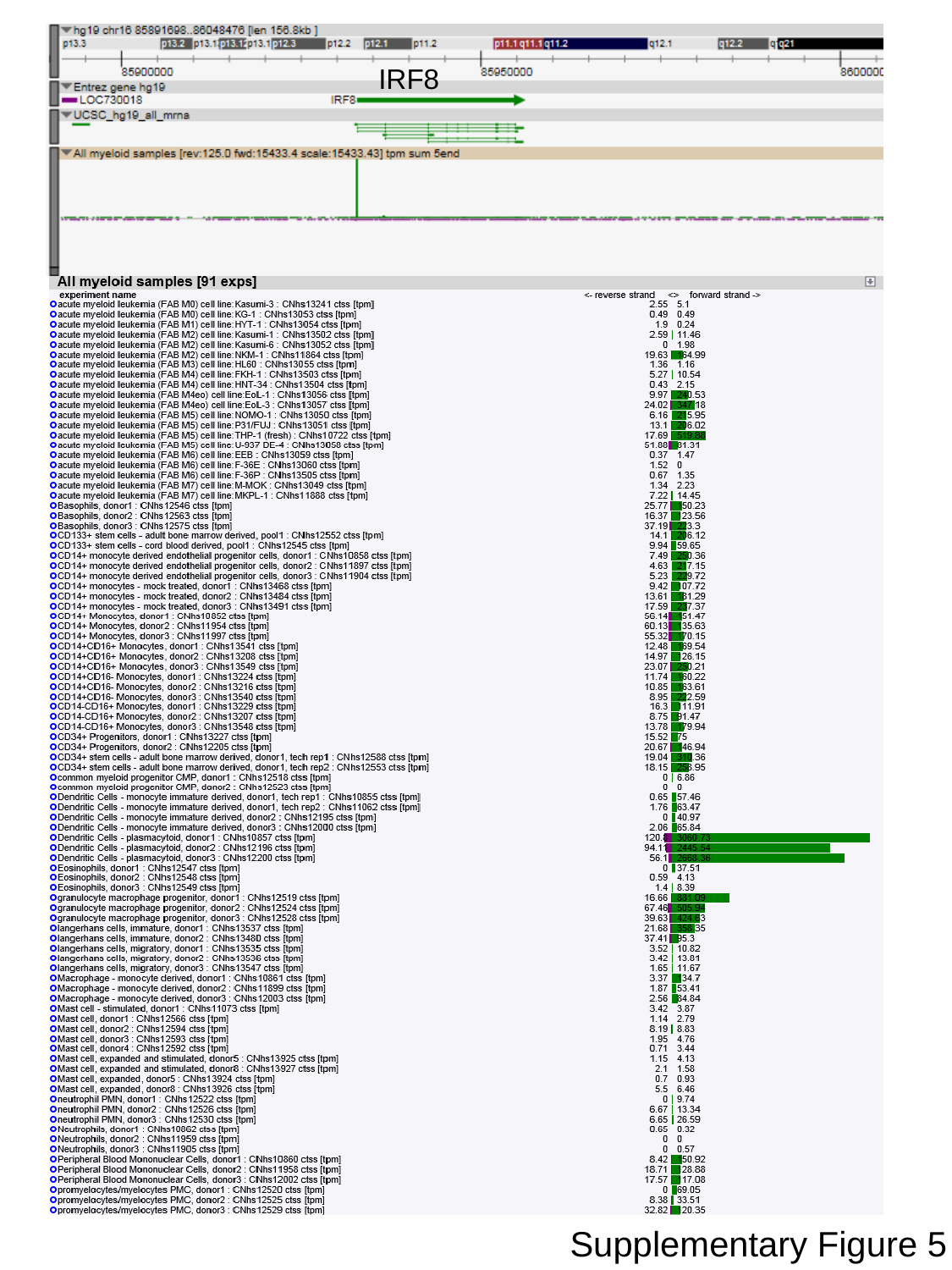

IRF8
Supplementary Figure 5

## Slide 6
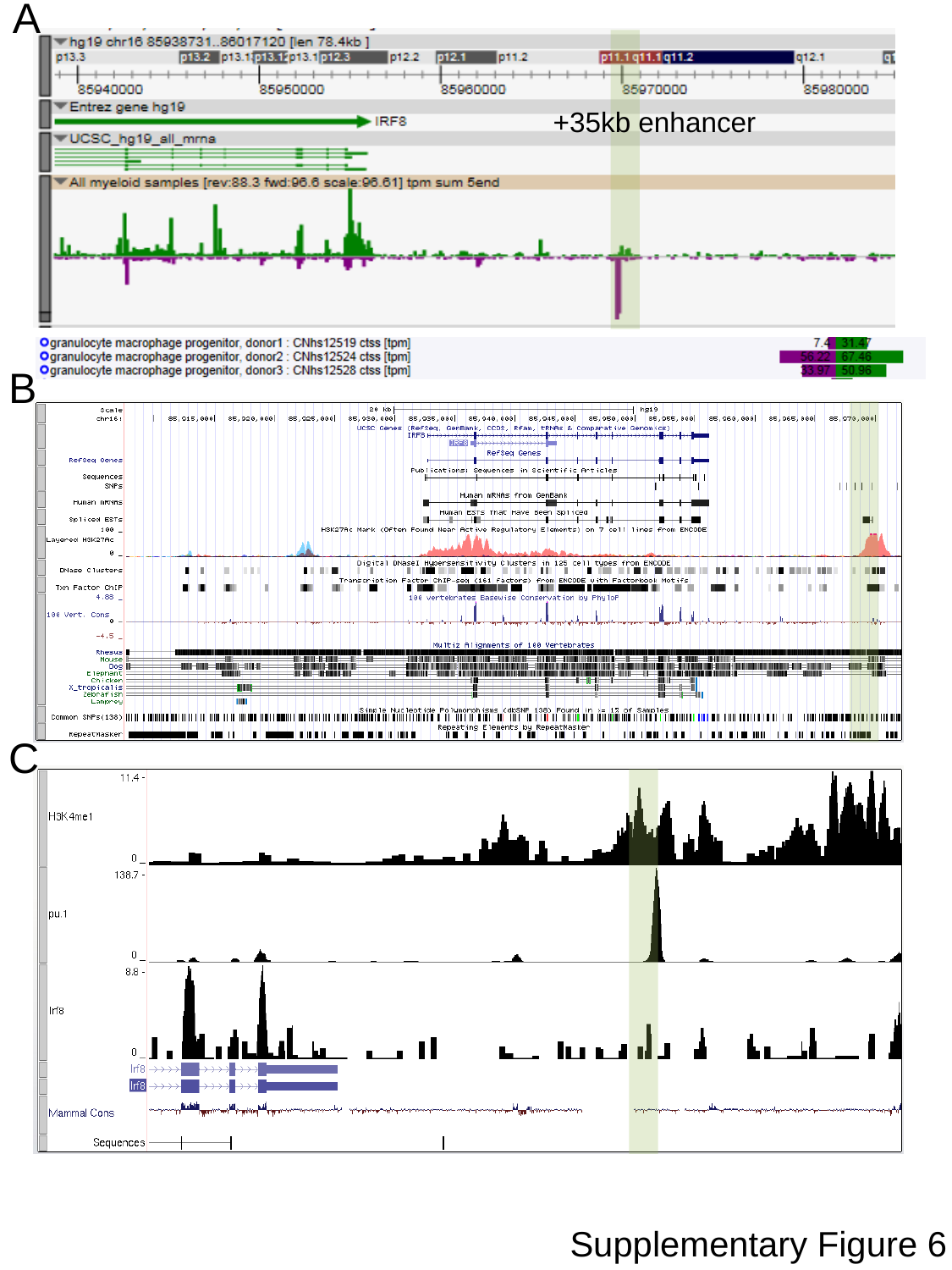

A
+35kb enhancer
B
C
Supplementary Figure 6

## Slide 7
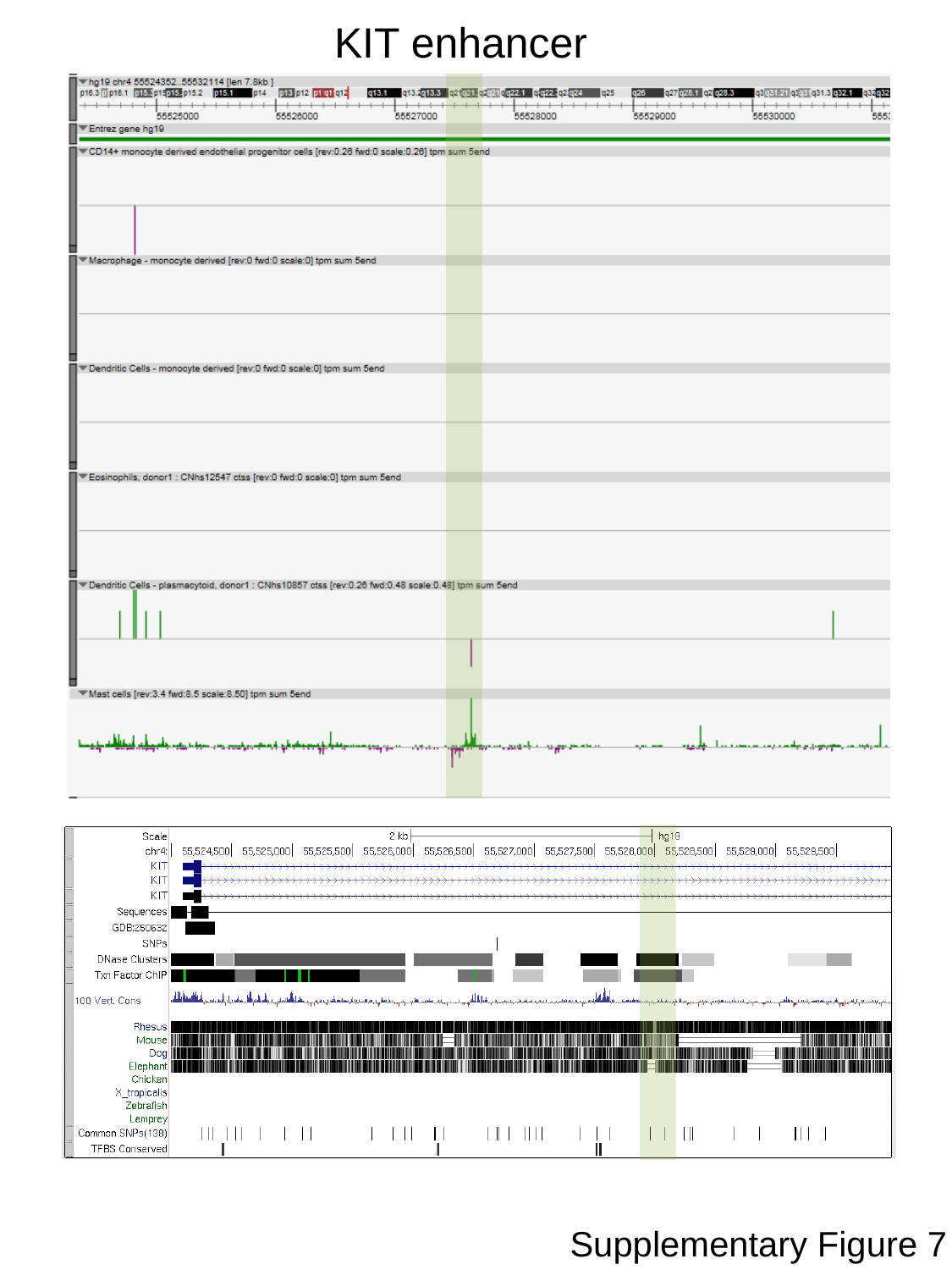

KIT enhancer
Supplementary Figure 7

## Slide 8
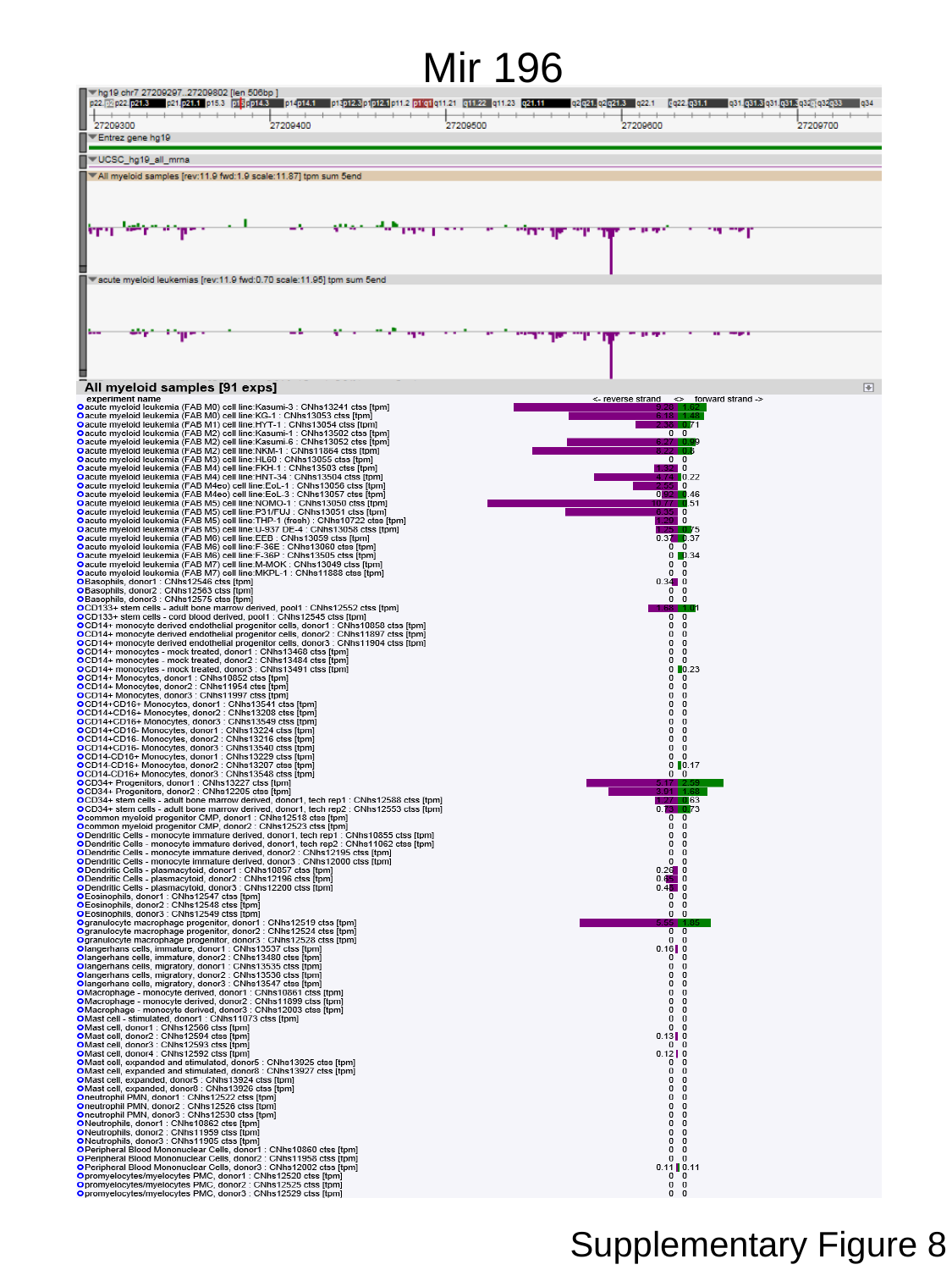

Mir 196
Supplementary Figure 8

## Slide 9
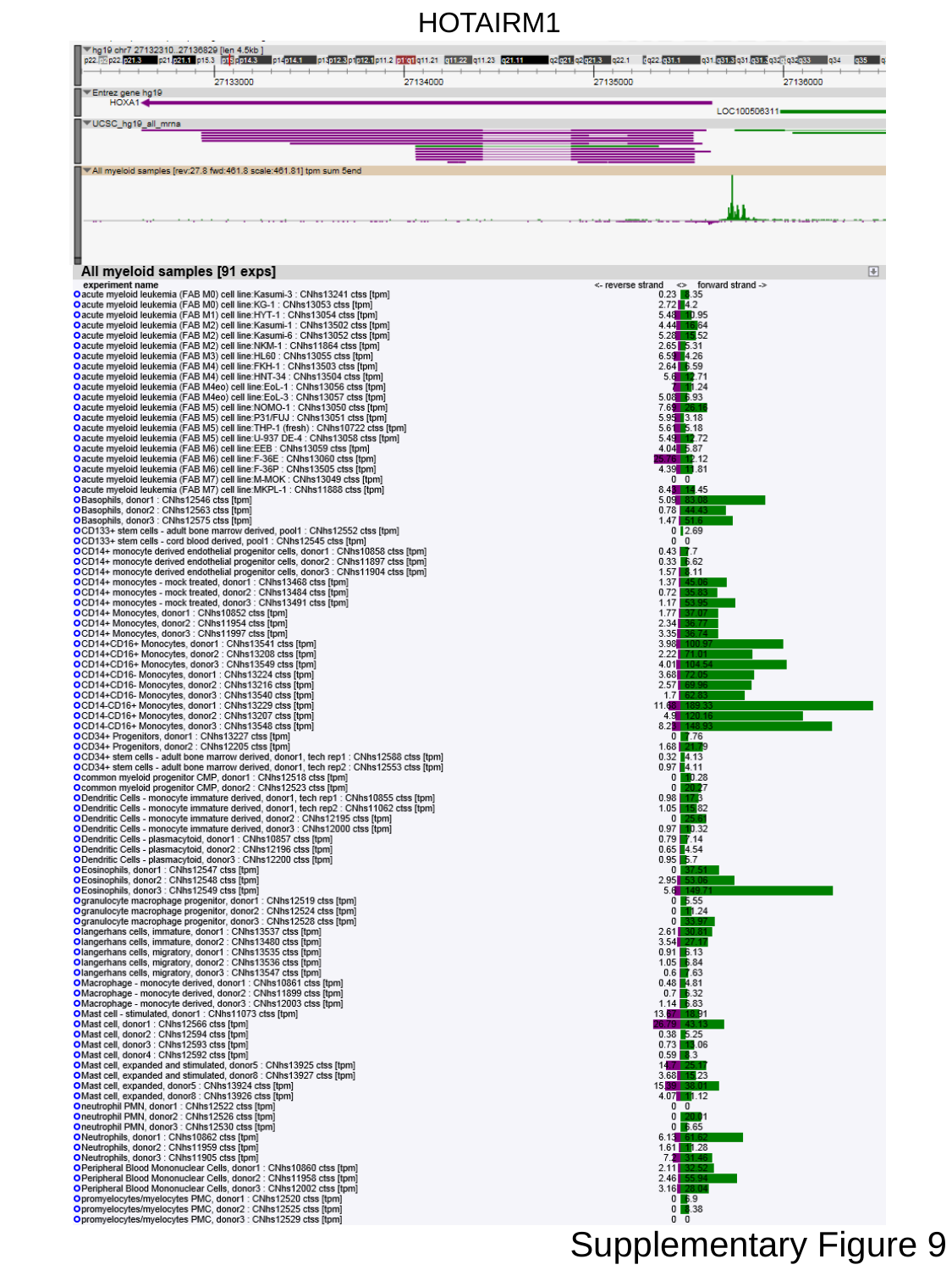

HOTAIRM1
Supplementary Figure 9

## Slide 10
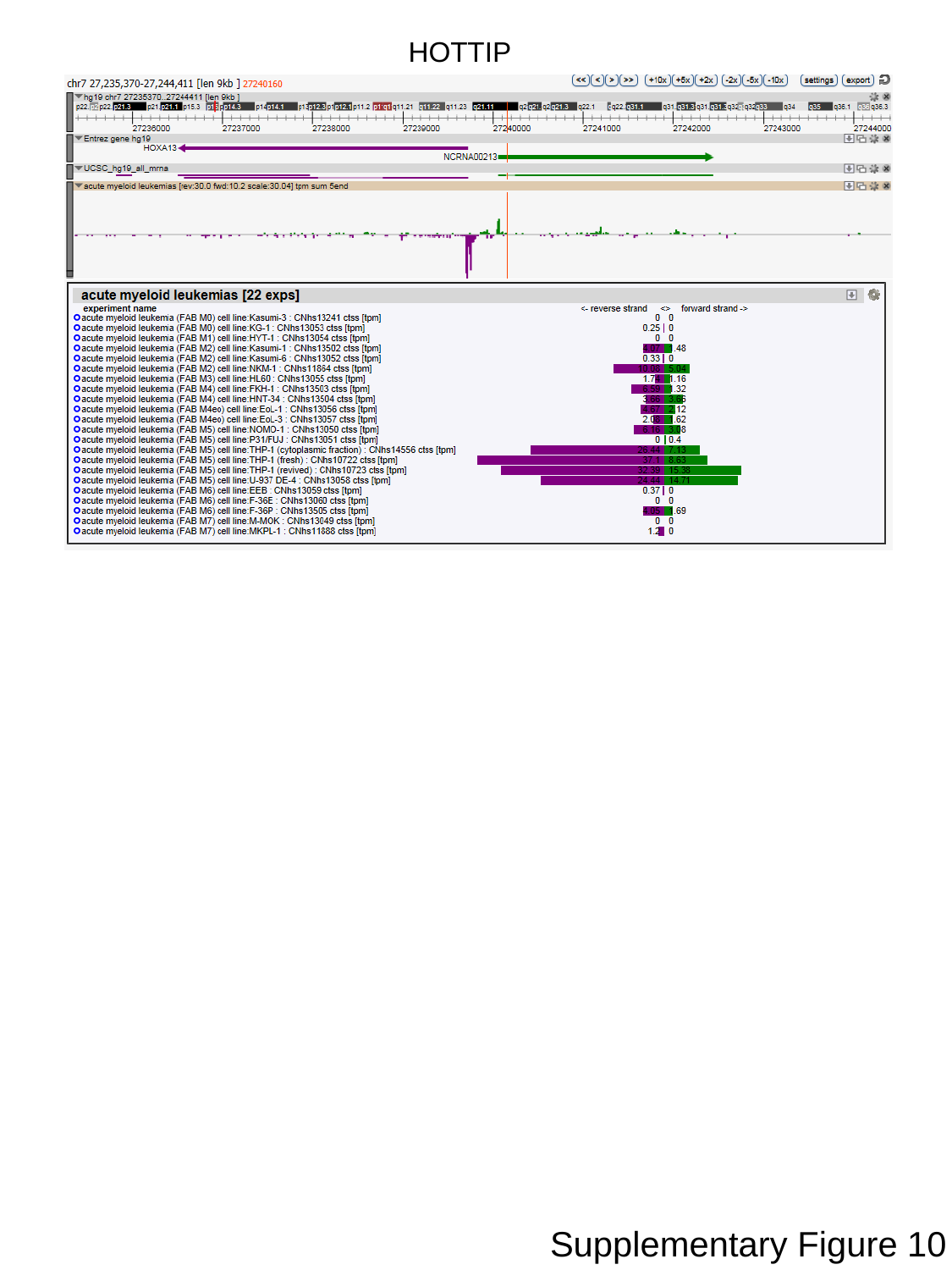

HOTTIP
Supplementary Figure 10

## Slide 11
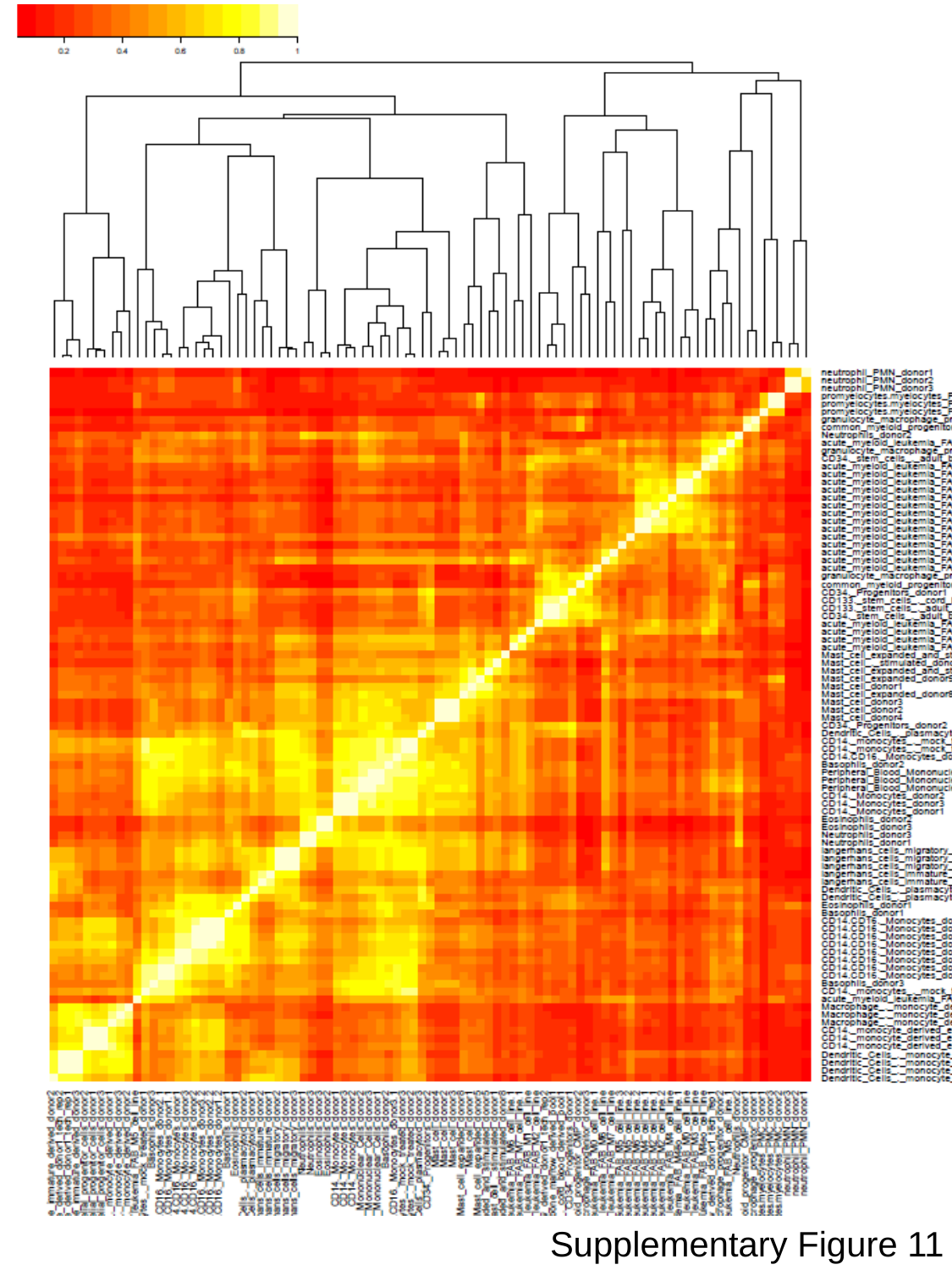

Supplementary Figure 11
