# Supplementary material for: Technical Advance: Transcription factor, promoter, and enhancer utilization in human myeloid cells
Source: J Leukoc Biol. 2015 Feb 25;97(5):985–95. doi: 10.1189/jlb.6TA1014-477RR (PMC4398258; doi:10.1189/jlb.6TA1014-477RR)
Supplement: Supplemental Data [file supp_jlb.6TA1014-477RR_Supplemental_Tables.docx]

Tables for

**Transcription factor, promoter and enhancer utilisation in human myeloid cells**

Anagha Joshi^1^¶, Christopher Pooley^1^, Tom Freeman^1^, Andreas Lennartsson^4^, Magda Babina^5^, Christian Schmidl^6^, Teunis Geijtenbeek^7^, the FANTOM consortium, Jessica Severin^3^, Masayoshi Itoh^3^, Timo Lassmann^3^, Hideya Kawaji^3^, Yoshihide Hayashizaki^2^, Harukazu Suzuki^3^, Alistair R. R. Forrest^3^, Michael Rehli^6^ and David Hume^1^¶

^1^The Roslin Institute and Royal (Dick) School of Veterinary Studies, The University of Edinburgh, Midlothian EH25 9RG, Scotland, United Kingdom

^2^RIKEN Preventive Medicine and Diagnosis Innovation Program, 1-7-22 Suehiro-cho, Tsurumi-ku, Yokohama, 230-0045 Japan.

^3^RIKEN Center for Life Science Technologies (Division of Genomic Technologies), 1-7-22 Suehiroo-cho, Tsurumi-ku, Yokohama, 230-0045 Japan.

^4^Department of Biosciences and Nutrition, Karolinska Institute, SE-14183 Huddinge, Sweden

^5^Department of Dermatology and Allergy, Charité Universitätsmedizin Berlin, Berlin, Germany

^6^Dept. Internal Medicine III, University Hospital, University of Regensburg, Germany

^7^Department of Experimental Immunology, Academic Medical Center, University of Amsterdam, Meibergdreef 9, 1105 AZ Amsterdam, the Netherlands.

¶To whom correspondence should be addressed

Running title: The Myeloid lineage transcriptome

**Table 1:** The details of all 91 samples including cell type, description, supplier, sex, age, number of peaks called**.** This table is provided as a separate excel spreadsheet.

Table 2: The list of cluster numbers and cell types in which genes in corresponding cluster are highly expressed in.

| Cluster | Highly expressing cell type |
| --- | --- |
| Cluster 1 | CD14+ monocytes |
| Cluster 2 | Eosinophils, Neutrophils |
| Cluster 3 | Neutrophils |
| Cluster 4 | Neutrophils |
| Cluster 5 | CD14+ monocytes, Neutrophils |
| Cluster 6 | Mast cells |
| Cluster 7 | Eosinophils |
| Cluster 8 | PMN neutrophils |
| Cluster 9 | Immature Langerhans cells |
| Cluster 10 | Mast cells |
| Cluster 11 | Plasmacytoid dendtritic cells |
| Cluster 12 | CMPs |
| Cluster 13 | Migratory Langerhans cells |
| Cluster 14 | CD14+ monocytes, Macrophages |
| Cluster 15 | AML FAB M4 |
| Cluster 16 | GMPs |
| Cluster 17 | promyelocytes |
| Cluster 18 | CD133+ stem cells |
| Cluster 19 | PMN neutrophils |
| Cluster 20 | Immature Langerhans cells |
| Cluster 21 | promyelocytes |
| Cluster 22 | PMN neutrophils |
| Cluster 24 | Eosinophils, Neutrophils |
| Cluster 25 | Dendritic cells |
| Cluster 26 | Mast cells |
| Cluster 28 | Eosinophils |
| Cluster 29 | AML FAB M6 |
| Cluster 30 | Mast cells |
| Cluster 31 | Macrophages |
| Cluster 32 | CD14+ monocytes |
| Cluster 33 | AML FAB M7 |
| Cluster 34 | Mast cells |
| Cluster 35 | Dendritic cells |
| Cluster 36 | CD14- CD16+ monocytes |
| Cluster 37 | Eosinophils, Neutrophils |
| Cluster 39 | CD14+ monocytes |
| Cluster 40 | AML FAB M6 |
| Cluster 41 | AML FAB M1 |
| Cluster 44 | Dendritic cells |
| Cluster 45 | AML FAB M6 |
| Cluster 46 | AML FAB M2 |
| Cluster 47 | AML FAB M5 |
| Cluster 48 | AML FAB M5 |
| Cluster 49 | Basophils, Eosinophils |
| Cluster 50 | AML FAB M7 |
| Cluster 53 | Mast cells |
| Cluster 55 | Basophils |
| Cluster 59 | Eosinophils |
| Cluster 60 | Mast cells |
| Cluster 63 | Macrophages, PMN neutrophils |
| Cluster 65 | PMN neutrophils |
| Cluster 68 | Langerhans cells |
| Cluster 73 | Mast cells |
| Cluster 74 | Dendritic cells, Macrophages |
| Cluster 81 | GMPs |
| Cluster 88 | GMPs, promyelocytes |
| Cluster 89 | CD14+ monocytes |
| Cluster 93 | Mast cells |
| Cluster 98 | PMN neutrophils |
| Cluster 103 | Mast cells |
| Cluster 106 | GMPs, promyelocytes |
| Cluster 107 | AML FAB M6 |
| Cluster 116 | Langerhans cells, Macrophages |
| Cluster 123 | Neutrophils |
| Cluster 124 | CD34+ progenitors |
| Cluster 126 | Dendritic cells |
| Cluster 132 | PMN neutrophils |
| Cluster 141 | Migratory Langerhans cells |
| Cluster 144 | Langerhans cells |
| Cluster 147 | Eosinophils |
| Cluster 160 | Basophils |
| Cluster 163 | AML FAB M5 |

Table 3: 10 enhancers overlapping with ENCODE high confidence enhancers^8^ along with their associated promoter predictions.

| Enhancer | Promoter | promoter name | Distance | correlation | P value | FDR |
| --- | --- | --- | --- | --- | --- | --- |
| chr8:141607900-141608150 | chr8:141608042..141608073,+ | p1@BC078139 | -38 | 0.991247 | 0 | 0 |
| chr10:3938329-3938538 | chr10:3819310..3819325,- | p5@KLF6 | -119125 | 0.800623 | 0 | 0 |
| chr10:3938329-3938538 | chr10:3827371..3827386,- | p2@KLF6 | -111064 | 0.756967 | 0 | 0 |
| chr1:211517513-211517976 | chr1:211500017..211500028,+ | p4@TRAF5 | 17695 | 0.640112 | 9.42E-12 | 4.46E-10 |
| chr6:143234736-143235083 | chr6:143266297..143266356,- | p1@HIVEP2 | 31297 | 0.636750 | 1.29E-11 | 6.03E-10 |
| chr10:3938329-3938538 | chr10:3818891..3818895,- | p6@KLF6 | -119544 | 0.601280 | 2.92E-10 | 1.15E-08 |
| chr8:141607900-141608150 | chr8:141645693..141645783,- | p1@EIF2C2 | 37688 | 0.586361 | 9.71E-10 | 3.58E-08 |
| chr1:211517513-211517976 | chr1:211665830..211665832,- | p5@RD3 | 148117 | 0.583994 | 1.17E-09 | 4.26E-08 |
| chr1:211517513-211517976 | chr1:211665365..211665411,- | p2@RD3 | 147652 | 0.577321 | 1.95E-09 | 6.91E-08 |
| chr7:92277080-92277232 | chr7:92462716..92462727,- | p43@CDK6 | 185587 | 0.576207 | 2.13E-09 | 7.48E-08 |
| chr20:5046140-5046913 | chr20:4680836..4680854,+ | p10@PRNP | 365921 | 0.563663 | 5.39E-09 | 1.79E-07 |
| chr10:3938329-3938538 | chr10:3823938..3823954,- | p13@KLF6 | -114497 | 0.536315 | 3.61E-08 | 1.06E-06 |
| chr10:3938329-3938538 | chr10:3827389..3827408,- | p1@KLF6 | -111046 | 0.532344 | 4.70E-08 | 1.36E-06 |
| chr5:142179452-142179898 | chr5:142149955..142150000,+ | p1@ARHGAP26 | 29886 | 0.528961 | 5.86E-08 | 1.67E-06 |
| chr7:92277080-92277232 | chr7:92463153..92463169,- | p8@CDK6 | 186024 | 0.524189 | 7.96E-08 | 2.23E-06 |
| chr7:92277080-92277232 | chr7:92465854..92465865,- | p6@CDK6 | 188725 | 0.51558 | 1.37E-07 | 3.70E-06 |
